# Histone lactylation enhances GCLC expression and thus promotes chemoresistance of colorectal cancer stem cells through inhibiting ferroptosis

**DOI:** 10.1038/s41419-025-07498-z

**Published:** 2025-03-20

**Authors:** Jiao Deng, Yangkun Li, Lanlan Yin, Shuang Liu, Yanqi Li, Wancheng Liao, Lei Mu, Xuelai Luo, Jichao Qin

**Affiliations:** 1 Molecular Medicine Center, Tongji Hospital, Tongji Medical College, Huazhong University of Science and Technology, Wuhan, Hubei China; 2 Department of Surgery, Tongji Hospital, Tongji Medical College, Huazhong University of Science and Technology, Wuhan, Hubei China; 3 Department of Gastrointestinal Surgery, Peking University Shenzhen Hospital, Shenzhen, Guangdong China; 4 Department of Gastrointestinal Surgery, the First Affiliated Hospital, Zhejiang University School of Medicine, Hangzhou, Zhejiang China

**Keywords:** Cancer stem cells, Cancer therapeutic resistance

## Abstract

Colorectal cancer stem cells (CCSCs) play a critical role in mediating chemoresistance. Lactylation is a post-translational modification induced by lactate that regulates gene expression. However, whether lactylation affects the chemoresistance of CCSCs remains unknown. Here, we demonstrate that histone lactylation enhances CCSC chemoresistance both in vitro and in vivo. Furthermore, our findings showed that p300 catalyzes the lactylation of histone H4 at K12, whereas HDAC1 facilitates its delactylation in CCSCs. Notably, lactylation at H4K12 (H4K12la) upregulates GCLC expression and inhibits ferroptosis in CCSCs, and the inhibition of p300 or LDHA decreases H4K12la levels, thereby increasing the chemosensitivity of CCSCs. Additionally, the GCLC inhibitor BSO promotes ferroptosis and sensitizes CCSCs to oxaliplatin. Taken together, these findings suggest that histone lactylation upregulates GCLC to inhibit ferroptosis signaling, thus enhancing CCSC chemoresistance. These findings provide new insights into the relationship between cellular metabolism and chemoresistance and suggest potential therapeutic strategies targeting p300, LDHA, and GCLC.

**Figure:**
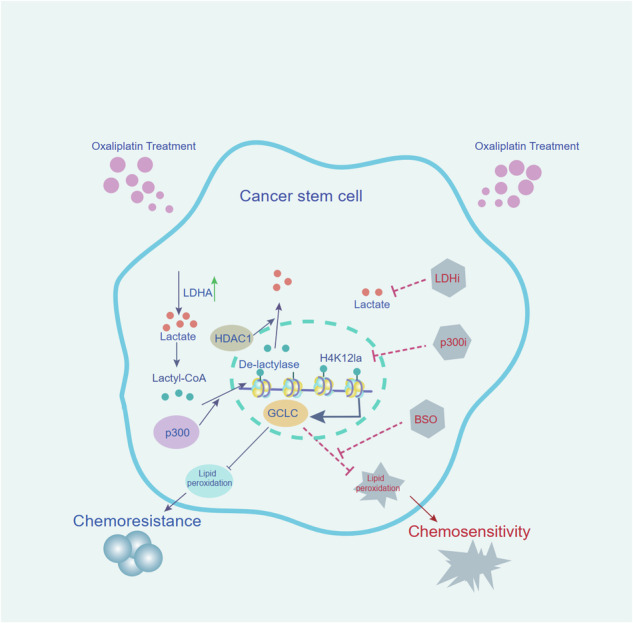
We showed that histones H4K12 lactylation promoted chemoresistance in CSCs. p300 catalyzes the lactylation of histone H4 at K12, HDAC1 inhibits the histone lactylation at the same site. H4K12la in CSCs regulates the expression of the ferroptosis-related gene GCLC, thereby inhibiting ferroptosis and leading to chemoresistance. Targeting the p300, LDHA, or GCLC may be overcome tumor chemoresistance.

We showed that histones H4K12 lactylation promoted chemoresistance in CSCs. p300 catalyzes the lactylation of histone H4 at K12, HDAC1 inhibits the histone lactylation at the same site. H4K12la in CSCs regulates the expression of the ferroptosis-related gene GCLC, thereby inhibiting ferroptosis and leading to chemoresistance. Targeting the p300, LDHA, or GCLC may be overcome tumor chemoresistance.

## Introduction

Lactate is a byproduct of aerobic glycolysis that promotes tumor cell proliferation, metastasis, and immunosuppression [1, 2]. However, its non-metabolic functions in cancer cells are not yet fully understood. In 2019, lactylation was identified as an epigenetic modification that regulates gene transcription through the modification of histone lysine residues [3, 4]. More recently, lactylation of both histone and non-histone lysine residues has been identified as prevalent post-translational modifications (PTMs), with dysregulation of lactylation linked to severe diseases, including cancer [5–8]. However, the role of lactylation in this pathological process remains largely unknown.

Colorectal cancer (CRC) contains a subpopulation of cells known as cancer stem cells (CSCs), which drive tumor growth, metastasis, and recurrence [9, 10]. Interactions within the tumor microenvironment (TME) have been found to play a significant role in chemoresistance [11]. Key features of the TME, such as hypoxia and acidic stress, regulate chemoresistance and enhance cancer cell stemness. Therefore, it is critical to understand the role of the TME in CRC progression and recurrence [12]. Lactate, a byproduct of tumor glycolysis, is a major contributor to TME acidification [13]. Therefore, insight into the mechanisms by which TME acidification drives chemoresistance in CSCs is essential for identifying novel therapeutic targets.

Ferroptosis is an iron-dependent, non-apoptotic form of cell death characterized by mitochondrial shrinkage and lipid peroxidation accumulation [14]. Recent studies have highlighted that targeting ferroptosis-associated proteins or employing ferroptosis inducers can effectively overcome chemoresistance in various tumor types [15–17]. Glutamate-cysteine ligase (GCLC) is a crucial enzyme in glutathione synthesis and plays a pivotal role in mitigating ferroptosis by inhibiting lipid peroxidation [18]. GCLC plays crucial roles in various tumor-associated biological processes, such as tumor proliferation, resistance to ferroptosis, chemoresistance, and immune evasion [19–22]. Metabolic abnormalities are a hallmark of cancer cells, with the Warburg effect being one of the most metabolic alterations. However, whether the Warburg effect inhibits ferroptosis by regulating GCLC expression, thereby contributing to chemoresistance in CSCs, remains unknown.

Herein, we show that histone lactylation enhances chemoresistance in CCSCs. Mechanistically, p300 catalyzes the lactylation of H4K12, whereas HDAC1 removes this modification at the same site. H4K12la in CCSCs regulates the ferroptosis-related gene GCLC, thereby suppressing ferroptosis and promoting chemoresistance. Furthermore, we enhanced the chemosensitivity of CCSCs by combining the GCLC inhibitor BSO with oxaliplatin. Taken together, these findings indicate that H4K12la suppresses ferroptosis in CCSCs. Targeting p300, LDHA, or GCLC may be an effective strategy to overcome chemoresistance in CRC.

## Results

### High levels of histone lactylation are associated with an unfavorable prognosis in colorectal cancer patients

**Fig. 1:**
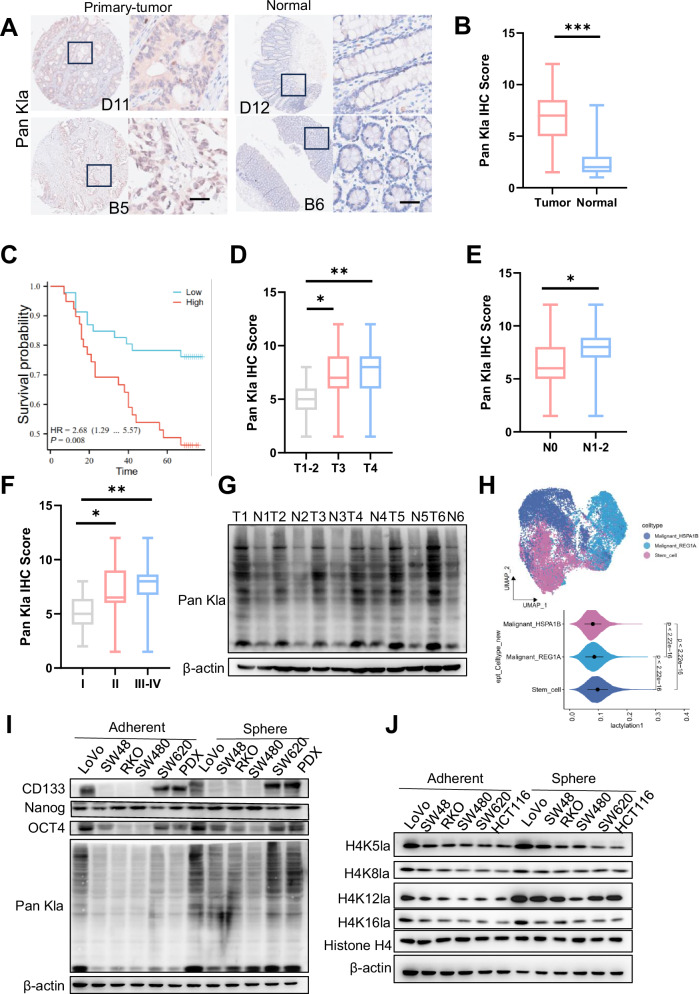
High levels of lactylation at histone are associated with an unfavorable prognosis in patients with colorectal cancer and increased histone lactylation in CCSCs. **A** Immunohistochemical (IHC) staining was used to test lactylation levels in both normal and primary CRC tumor tissues. Scale bar: 50 μm. **B** Statistical results of IHC scores for lactylation levels in normal and primary CRC tissues. **C** Kaplan-Meier curves of overall survival indicate a significant difference between patients with colorectal cancer expressing low and high lactylation levels. High: IHC score >7, *n* = 39; Low: IHC score ≤7, *n* = 49; *p* = 0.008. **D** Lactylation levels in primary colorectal cancer tissues at different T stages. 11 cases were in the T_1-2_ stage, 49 were in the T_3_ stage, and 28 were in the T_4_ stage. **E** Lactylation levels in primary colorectal cancer tissues at different N stages: 60 cases in the N_0_ stage and 28 cases in the N_1-2_ stage. **F** Lactation levels in primary colorectal cancer tissues at different TNM stages. 10 patients had I stage, 48 had II stage, and 30 had stages III and IV. **G** western blotting was used to evaluate the expression of Pan Kla in tumor and normal specimens; T, tumor; N, normal. **H** The UMAP plot of clustered single-cell RNA-seq datasets demonstrates the existence of distinct CRC subpopulations. The cells were clustered into cancer stem cells and other cancer cells, with each cell cluster labeled and colored according to its subcell type. Density distribution of the lactylation score in tumor subpopulations. The lactylation score signatures were calculated from the average relative expression level of a key lactylation-related gene. **I** Western blot analysis was used to evaluate the expression of CD133, Pan Kla, Nanog, and OCT4 in CRC sphere and adherent cells. **J** Western blot analysis was performed to evaluate the expression of H4K5la, H4K8la, H4K12la, and H4K16la in CRC spheres and adherent cells. Three biological replicates are shown. The data represent the mean ± SD. In (**B**) and (**E**), comparisons were made using unpaired Student’s t-test, whereas in (**D**) and (**F**), comparisons were made using One-way ANOVA with Tukey’s test. * *p* < 0.05, **p < 0.01.

Lactylation is a recently discovered PTM whose role in the cellular processes remains largely unknown. Colon cancer exhibits active glycolysis (Supplementary Fig. S1A), leading to the production of substantial amounts of lactate, which serves as a substrate for lactylation. To explore the clinical significance of protein lactylation in colorectal carcinogenesis and progression, we analyzed its levels in colorectal cancer tissues. Immunohistochemistry (IHC) staining was performed on 88 colorectal cancer tissues and 81 normal colorectal tissues, revealing significantly elevated global lactylation levels in tumor tissues compared to normal tissues (*p* < 0.05) (Fig. 1A, B and Supplementary Fig. S1B). In addition, the results showed that elevated lactylation levels were negatively correlated with overall patient survival (Fig. 1C). We found that lactylation scores from IHC staining were positively correlated with T (primary tumor), N (node), and clinical stages (Fig. 1D–F). Next, we collected 12 pairs of primary colon cancer tissues and their adjacent non-cancerous tissues. Western blot analysis showed that global lactylation levels were higher in cancerous tissues than in non-cancerous tissues (Fig. 1G and Supplementary Fig. S1C). CSCs are a key driver of tumor recurrence and drug resistance [23]. However, the effects of lactylation on CSCs remain unclear. We conducted a detailed analysis of the gene expression profiles of specific cellular components within colorectal cancer tissues using single-cell transcriptomics. Analysis of GSE205506 and GSE245552 identified eight cell clusters (Supplementary Fig. S1D). To quantitatively evaluate lactylation in each cell type, we defined the “lactylation score” across all single cells, calculated as the average relative expression of lactylation-related genes in CRC (Table S8). Notably, CCSCs exhibited high lactylation score signatures (Fig. 1H). To investigate the role of lactylation in CCSCs, we compared the lactylation levels in CSCs (sphere cells) with those in cancer-differentiated cells (CDCs) (adherent cells). Western blotting revealed that the overall lactylation levels were higher in sphere cells than in adherent cells (Fig. 1I and Supplementary Fig. S1E). The main band detected by western blot had a molecular weight of approximately 15 kDa. Mass spectrometry (MS) analysis was conducted after immunoprecipitation of lactylated proteins. These results indicated that the band predominantly consisted of histone H4 (Table S1). Indeed, western blot analysis of histone H4 lactylation showed a significant increase in H4K12la levels in CSCs compared to CDCs (Fig. 1J). Moreover, H4K12la levels were elevated in colorectal cancer tissues relative to normal colorectal tissues (Supplementary Fig. S1F). Finally, CRC sphere cells were incubated with an anti-CD133 antibody and sorted into CD133^+^ and CD133^−^ cells using flow cytometry. Western blotting assays demonstrated higher lactylation in CD133^+^ cells than in CD133^-^ cells (Supplementary Fig. S1G, H). These results suggest that lactylation is generally increased in colorectal cancer tissues and that the histone lactylation levels are higher in CCSCs than CCDCs.

### Lactate-regulated histone Kla in cancer stem cells

**Fig. 2:**
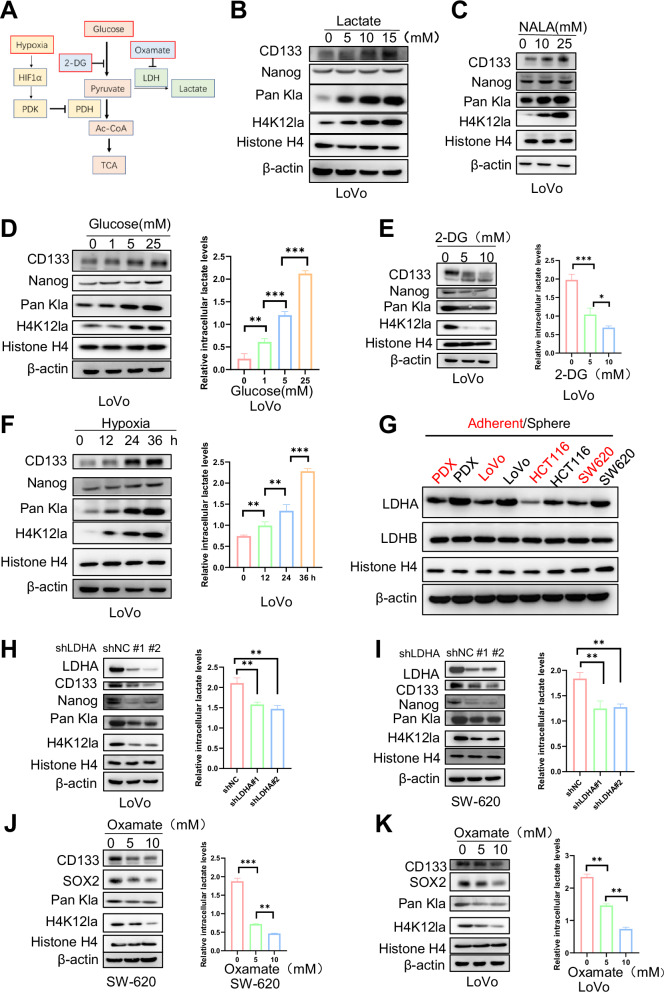
Lactate regulates histone lactylation and stemness of cancer stem cells. **A** Various metabolic modulators regulate glycolysis and lactate production. **B** Western blot analysis was performed to test the expression of CD133, Pan Kla, Nanog, and H4K12la in LoVo CSCs cultured in varying lactate concentrations for 24 h. **C** Western blot analysis was performed to evaluate the expression of CD133, Pan Kla, Nanog, and H4K12la in LoVo CSCs cultured in varying NALA concentrations for 24 h. **D** Western blot analysis was used to evaluate the expression of CD133, Nanog, Pan Kla, and H4K12la in LoVo CSCs cultured in varying glucose concentrations for 24 h; Right: Intracellular lactate levels were measured. **E** LoVo CSCs were exposed to varying concentrations of 2-DG for 24 h. Left: Western blot analysis was used to assess the expression of CD133, Nanog, Pan Kla, and H4K12la; Right: Intracellular lactate levels were measured. **F** LoVo CSCs were cultured under hypoxic conditions for different durations. Left: Western blot analysis was used to evaluate the expression of CD133, Nanog, Pan Kla, and H4K12la; Right: Intracellular lactate levels were measured. **G** Western blot analysis was performed to test the expression of LDHA and LDHB in CRC sphere cells and adherent cells. **H** Two short hairpin RNAs of LDHA were used to knock down the expression of LDHA in LoVo CSCs. Left: Western blot analysis was used to evaluate the expression of LDHA, CD133, Nanog, Pan Kla, and H4K12la; Right: Intracellular lactate levels were measured. **I** Two short hairpin RNAs of LDHA were used to knock down the expression of LDHA in SW620 CSCs. Left: Western blot analysis was used to assess the expression of LDHA, CD133, Nanog, Pan Kla, and H4K12la; Right: Intracellular lactate levels were measured. **J** SW620 CSCs were exposed to varying concentrations of oxamate for 24 h. Left: Western blot analysis was used to evaluate the expression of CD133, Nanog, Pan Kla, and H4K12la; Right: Intracellular lactate levels were measured. **K** LoVo CSCs were exposed to varying concentrations of oxamate for 24 h. Left: Western blot analysis was used to evaluate the expression of CD133, Nanog, Pan Kla, and H4K12la; Right: Intracellular lactate levels. Three biological replicates were shown. Comparisons were conducted using one-way ANOVA with Tukey’s test. *p < 0.05, **p < 0.01, ***p < 0.001.

As previous studies have shown that both intra- and extracellular lactate drive lysine lactylation (Kla) on histones [3], we tested whether extrinsic and intracellular lactate affect the Pan Kla levels in CCSCs (Fig. 2A). CCSCs were exposed to various concentrations of lactate or sodium lactate (NALA). The results demonstrated that Pan Kla and H4K12la levels increased in a dose-dependent manner (Fig. 2B, C and Supplementary Fig. S2A, B). Furthermore, immunofluorescence (IF) analysis showed that lactate treatment resulted in increased H4K12la levels in CCSCs compared to the control group (Supplementary Fig. S2D). Notably, lactate levels, as well as Pan Kla and H4K12la levels, increased in a dose-dependent manner in CCSCs incubated with glucose (Fig. 2D and Supplementary Fig. S2C). In contrast, treatment of CCSCs with 2-deoxy-D-glucose (2-DG), a non-metabolizable glucose analog [3], led to a decrease in intracellular lactate, Pan Kla, and H4K12la levels (Fig. 2E and Supplementary Fig. S2E). Consistent with previous findings [3], hypoxia promoted lactate production and elevated Pan Kla and H4K12la levels in CCSCs (Fig. 2F and Supplementary Fig. S2F). To test whether LDHA/B mediates lactate metabolism in CCSCs, we performed Western blot analysis on both adherent and sphere CRC cells. Our findings indicated that LDHA expression was significantly higher in CCSCs than in CCDCs (Fig. 2G). Notably, when CCSCs were knocked down with shRNA or treated with oxamate, an LDHA inhibitor (LDHi) [3], Pan Kla, H4K12la, and intracellular lactate levels decreased (Fig. 2H–K). Importantly, we analyzed the correlation between LDHA expression levels and patient prognosis in colorectal cancer using the TCGA database. Kaplan-Meier survival analysis indicated that low LDHA expression was correlated with prolonged overall survival (Supplementary Fig. S2G, H). In conclusion, these findings suggest that lactate mediates H4K12la levels in CCSCs.

### H4K12la is catalyzed by p300 and abrogated by HDAC1

**Fig. 3:**
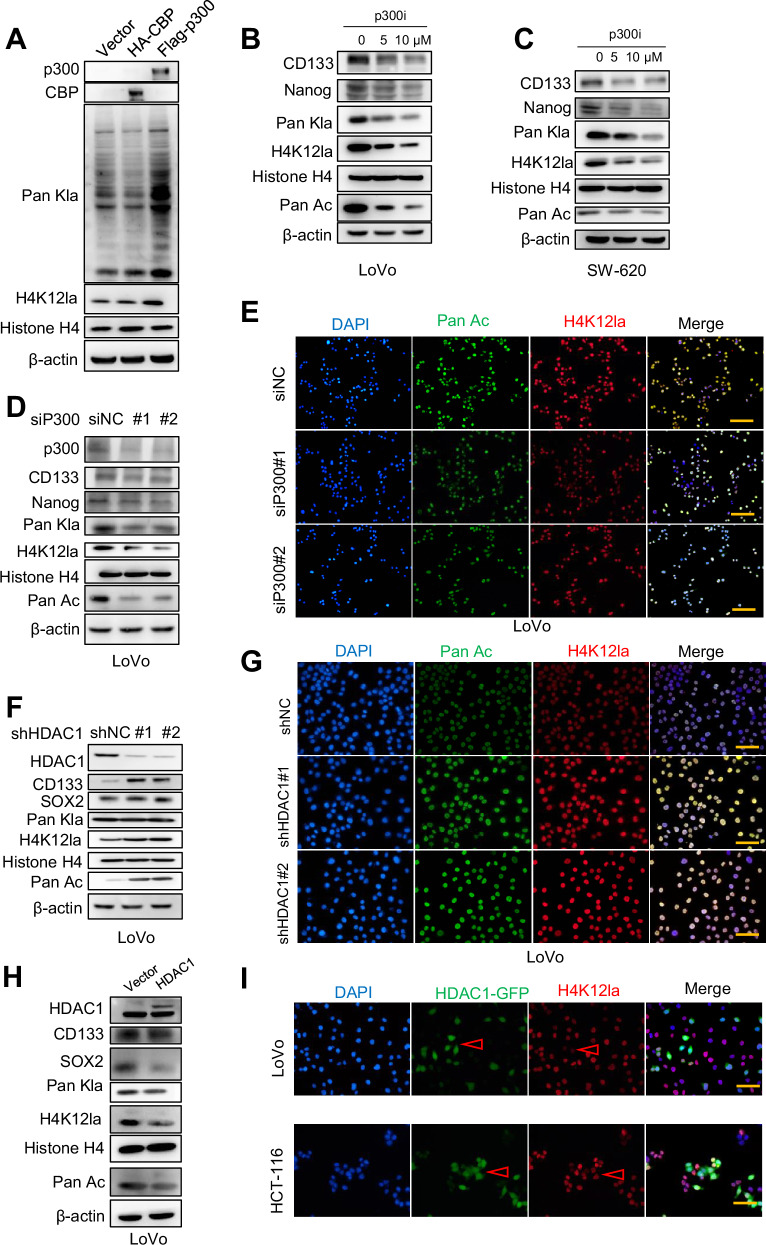
H4K12 is lactylated by p300 and de-lactylated by HDAC1. **A** Western blot analysis was used to evaluate the expression of p300, CBP, Pan Kla, and H4K12la in LoVo CSCs transfected with the Vector, HA-CBP, Flag-p300. **B**, **C** Western blot analysis was performed to assess the expression of CD133, Nanog, Pan Kla, H4K12la, and Pan Ac in LoVo and SW-620 CSCs cultured for 24 h with different concentrations of p300 inhibitor. **D** Western blot analysis was performed to evaluate the expression of p300, CD133, Nanog, Pan Kla, H4K12la, and Pan Ac in siP300 LoVo CSCs. **E** Immunofluorescence (IF) assay was performed to assess the expression of H4K12la and Pan Ac in response to p300 Scale bar: 200 μm. **F** Western blot analysis was used to evaluate the expression of the indicated molecules in shHDAC1 LoVo CSCs. **G** IF assay was performed to evaluate the expression of H4K12la and Pan Ac upon HDAC1 knockdown. Scale bar: 100 μm. **H** Western blot analysis was performed to evaluate the expression of the indicated molecules in LoVo CSCs overexpressing HDAC1. **I** IF assay of HCT-116 and LoVo sphere cells overexpressed HDAC1-GFP to assess H4K12la levels. The arrowhead indicates the cells overexpressing HDAC1 and under expressing H4K12la. Scale bar: 100 μm.

To identify the lactyltransferases responsible for lactylation at H4K12 in CCSCs, we examined the physical interactions of the acetyltransferases p300 and CBP with Pan Kla that were observed in the Pan Kla-binding protein profile (Table S1). Additionally, we found that HDAC1, a delactylase [24], physically interacts with Pan Kla (Table S1). To investigate the role of p300 and CBP as lactyltransferases in CCSCs, we overexpressed these proteins in CCSCs. The results showed an increase in Pan Kla and H4K12la levels in cells overexpressing p300 (Fig. 3A). To further investigate whether p300 mediates histone lactylation in CCSCs, p300 was knocked down by small interfering RNA (siRNA) in CCSCs, as confirmed by Western blot analysis. p300 knockdown significantly reduced Pan Kla and H4K12la levels (Fig. 3D and Supplementary Fig. S3A). Immunofluorescence (IF) analysis showed that siP300 also decreased Pan Acetylation (Pan Ac) and H4K12la levels in CCSCs (Fig. 3E and Supplementary Fig. S3B). Consistent with the above findings, treatment with a p300 inhibitor (p300i) similarly reduced Pan Kla and H4K12la levels in CCSCs (Fig. 3B, C). HDAC1 has been shown to have delactylase activity in cells [24]. We next explored the role of HDAC1 in histone lactylation. Western blot analysis revealed that HDAC1 knockdown using shRNA or treatment with the HDAC1 inhibitor TSA increased H4K12la levels (Fig. 3F and Supplementary Fig. S3C, E). IF also demonstrated that HDAC1 knockdown increased Pan Ac and H4K12la levels (Fig. 3G and Supplementary Fig. S3D). Conversely, HDAC1 overexpression reduced Pan Kla and H4K12la levels, as confirmed by Western blot and IF (Fig. 3H, I and Supplementary Fig. S3F). These findings suggest that H4K12la may be lactylated by p300 and delactylated by HDAC1 in CCSCs.

### H4K12la promotes chemoresistance in CCSCs

**Fig. 4:**
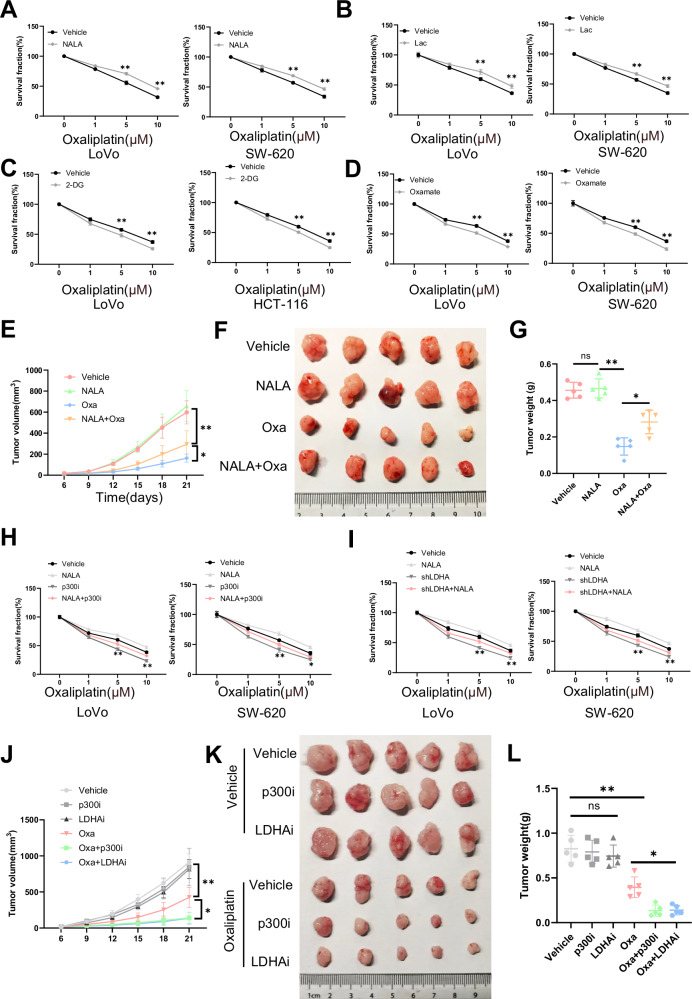
Inhibition of histone lactylation sensitizes CCSCs to oxaliplatin. **A** Cell survival was evaluated in LoVo and SW-620 CSCs treated with NALA plus the indicated doses of oxaliplatin for 24 h, vehicle treatment as negative control. **B** Cell survival was assessed in LoVo and SW-620 CSCs treated with lactate plus oxaliplatin for 24 h, with vehicle treatment as negative control. **C** Cell survival was tested in HCT-116 and LoVo CSCs treated with 2-DG plus oxaliplatin for 24 h, with vehicle treatment as negative control. **D** Cell survival was assessed in LoVo and SW-620 CSCs treated with oxamate plus oxaliplatin for 24 h. **E**–**G** Nude mice were injected subcutaneously with LoVo CSCs and then received the injection oxaliplatin (Oxa) at 10 mg/kg twice a week, and NALA at 120 mg/kg once a day, with vehicle injection as negative control. Tumor volumes were measured every 3 days (*n* = 5), growth curves were plotted (**E**), harvested tumors were photographed (**F**), and tumor weights (**G**) were measured (*n* = 5). **H**, **I** Cell survival was tested in SW-620 and LoVo CSCs treated with the indicated treatments and oxaliplatin for 24 h. **J**–**L** Nude mice were injected subcutaneously with LoVo CSCs and then treated with oxaliplatin (Oxa) at 10 mg/kg twice a week, along with either LDHi at 1000 mg/kg or p300i at 15 mg/kg every two days, respectively. Tumor volumes were measured every 3 days (*n* = 5) (**J**), harvested tumors were photographed (**K**), and weights were measured (*n* = 5) (**L**). Three biological replicates were shown. The data shown represent the mean ± SD. Statistical analyses were performed using the Student’s t-test. *p < 0.05; **p < 0.01; ***p < 0.001; ns stands for no significant differences.

CSCs exhibit significant resistance to chemotherapy and have been identified as the primary contributors to chemotherapy failure [25]. To investigate whether histone lactylation in CCSCs mediates the chemotherapeutic response of CRC cells, we used oxaliplatin, which is one of the most commonly used drugs in colorectal chemotherapy [26], to test the chemoresistance of CCSCs upon treatment with lactate and NALA. The results showed that CCSCs were more resistant to oxaliplatin upon administration of either NALA or lactate (Fig. 4A, B). CCSCs cultured with 25 mM glucose were more resistant to oxaliplatin than those cultured with 10 mM glucose (Supplementary Fig. S4A). More importantly, CCSCs became more sensitive to oxaliplatin when treated with 2-DG and oxamate (Fig. 4C, D). To examine whether lactylation mediates chemoresistance in vivo, we subcutaneously injected LoVo sphere cells into nude mice. The results demonstrated that the tumors in mice treated with NALA plus oxaliplatin grew larger than those treated with oxaliplatin alone (Fig. 4E–G). Immunohistochemical (IHC) staining for H4K12la and Ki67 confirmed these findings (Supplementary Fig. S4B). These findings indicate that lactylation plays a role in modulating chemoresistance in CCSCs. To further explore the potential of targeting lactylation as a strategy to overcome chemoresistance, we investigated the effects of inhibiting histone lactylation on CCSC viability. The results showed that p300i decreased the viability of CCSCs, whereas NALA plus p300i increased the viability (Fig. 4H). Moreover, our results showed that shLDHA decreased the viability of CCSCs in response to oxaliplatin, whereas NALA treatment increased cell survival (Fig. 4I). To further assess the role of lactylation inhibition in CRC chemotherapy in vivo, sphere-derived LoVo cells were subcutaneously injected into nude mice. The results demonstrated that tumors treated with p300i or LDHi grew more slowly during chemotherapy than those in the control group (Fig. 4J-L). IHC staining for H4K12la and Ki67 confirmed these findings (Supplementary Fig. S4C). These data clearly suggest that the inhibition of histone lactylation can decrease the chemoresistance of CCSCs.

### H4K12 lactylation inhibits ferroptosis in CCSCs

**Fig. 5:**
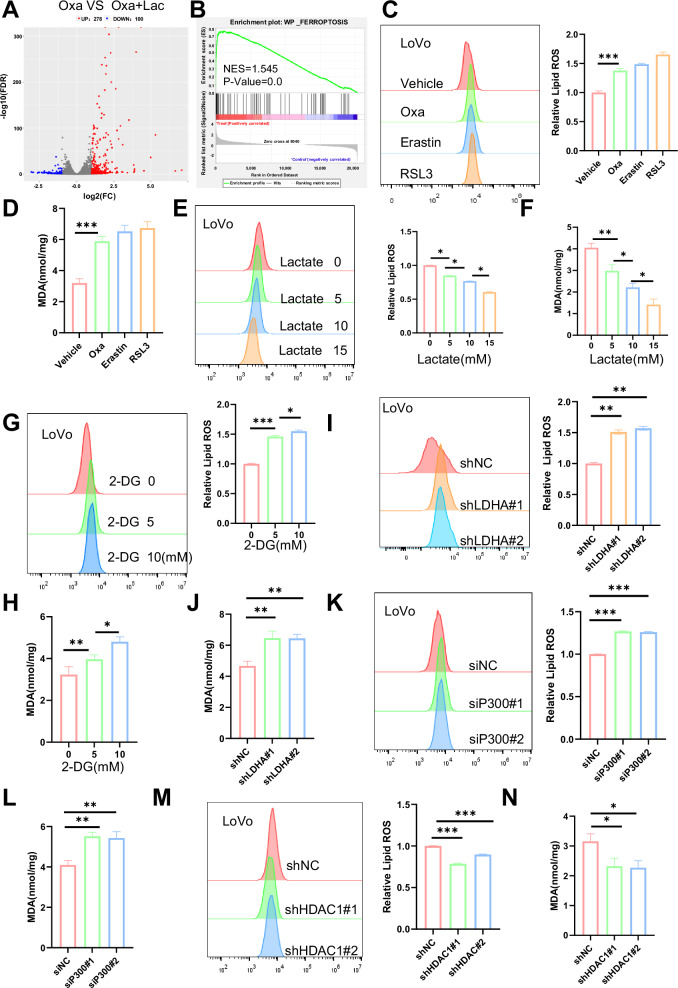
Lactylation of histones inhibits ferroptosis in CCSCs. **A** Volcano plots displaying differential RNA expression in LoVo CSCs treated with oxaliplatin or oxaliplatin plus lactate. Genes that were identified as differentially expressed by DESeq2 (Wald test) are shown in red and blue, with 278 upregulated and 100 downregulated genes. Oxa: oxaliplatin; Lac: lactate. **B** Gene Set Enrichment analysis (GSEA) of bulk RNA-seq was performed using the dataset. Representative GSEA plots indicate the enrichment of ferroptosis-related genes in the WikiPathways (WP) cancer gene sets (NES = 1.545, *p* < 0.0). NES, normalized enrichment score; *p*-value, normalized *p*-value. **C**, **D** Relative lipid ROS and malondialdehyde (MDA) levels were assayed in the LoVo CSCs treated with RSL3 (5 μM) or erastin (5 μM) or oxaliplatin(5 μM) for 12 h (*n* = 3). **E**, **F** Relative levels of lipid ROS and MDA were measured in LoVo CSCs treated with oxaliplatin(5 μM) and lactate for 12 h (*n* = 3). **G**, **H** Relative levels of lipid ROS and MDA were measured in LoVo CSCs treated with oxaliplatin(5 μM) and 2-DG for 12 h (*n* = 3). **I**, **J** Relative lipid ROS and MDA levels were measured in shNC and shLDHA LoVo CSCs treated with oxaliplatin (5 μM) for 12 h (*n* = 3). **K**, **L** Relative lipid ROS and MDA levels were measured in siNC and siP300 LoVo CSCs treated with oxaliplatin (5 μM) for 12 h (*n* = 3). **M**, **N** Relative lipid ROS and MDA levels were measured in shNC and shHDAC1 LoVo CSCs treated with oxaliplatin (5 μM) for 12 h (*n* = 3). Three biological replicates were shown. The presented data show the mean ± SD. Comparisons were conducted using one-way ANOVA with Tukey’s test. *p < 0.05, **p < 0.01, ***p < 0.001.

To identify genes involved in histone lactylation and chemoresistance in CCSCs, we performed RNA-seq analysis on LoVo CSCs treated with oxaliplatin with or without lactate. Differential gene expression analysis, visualized through volcano plots and heatmaps (Fig. 5A and Supplementary Fig. S5A), showed that in cells treated with both oxaliplatin and lactate, 278 genes were upregulated and 100 genes were downregulated, compared to cells treated with oxaliplatin alone. GSEA enrichment and KEGG signaling pathway analysis (Fig. 5B and Supplementary Fig. S5B) showed that lactylation may inhibit ferroptosis signaling, contributing to chemoresistance in CCSCs. To further investigate whether oxaliplatin induces ferroptosis in CCSCs, we quantified lipid ROS and malondialdehyde (MDA) levels in CCSCs treated with oxaliplatin, or the ferroptosis inducers RSL3 and Erastin. The results showed that oxaliplatin-treated CCSCs exhibited increased levels of both lipid ROS and MDA, suggesting that ferroptosis induction is regulated by lactylation in CCSCs. (Fig. 5C, D and Supplementary Fig. S5C, D). To explore whether lactylation mediates oxaliplatin-induced ferroptosis in CCSCs, we treated these cells with varying concentrations of lactate or NALA. This treatment decreased lipid ROS and MDA levels (Fig. 5E, F and Supplementary Fig. S5E-J). More importantly, treatment with 2-DG increased in lipid ROS and MDA levels in CCSCs (Fig. 5G, H and Supplementary Fig. S5K, L). To further investigate whether histone lactylation mediates oxaliplatin-induced ferroptosis in CCSCs, shLDHA, and siP300 CCSCs were treated with oxaliplatin. The results showed that these cells exhibited higher lipid ROS and MDA levels (Fig. 5I–L and Supplementary Fig. S6A–D). Additionally, HDAC1 knockdown reduced lipid ROS and MDA levels in oxaliplatin-treated CCSCs (Fig. 5M, N and Supplementary Fig. S6E, F). Collectively, these findings indicate that H4K12la in CCSCs may play a crucial role in chemoresistance by inhibiting the ferroptosis signaling pathway.

### Identification of potential downstream targets of the H4K12la

**Fig. 6:**
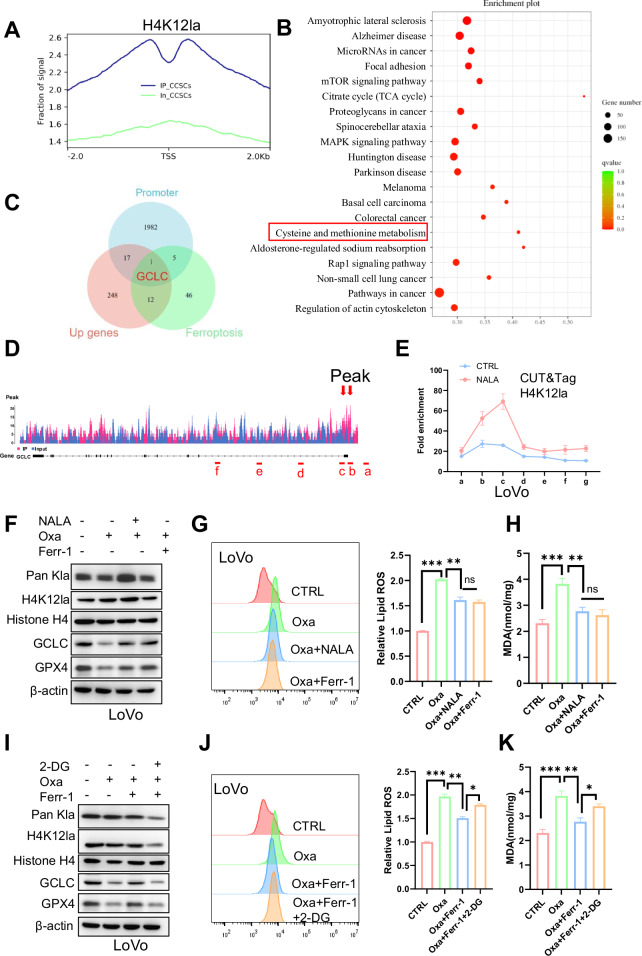
Histone lactyation transcriptionally activates expression of GCLC. **A** Distribution of H4K12la sites relative to the translation start site (TSS). **B** KEGG pathway analysis of H4K12la peaks. **C** Bioinformatic analysis revealed that GCLC is a downstream target of H4K12la. **D** IGV tracks for GCLC were generated using ChIP-seq analysis. Sites a-f are distributed across the GCLC genomic region, with sites b and c corresponding to H4K12la peaks. **E** CUT&Tag-qPCR assay was used to analyze the status of H4K12la in the GCLC genomic region in LoVo CSCs. **F** Western blot was performed to evaluate the expression of GCLC, Pan-Kla, H4K12la, and GPX4 in LoVo CSCs treated with NALA (10 μM) or Ferr-1(1 μM) plus oxaliplatin (Oxa). **G**, **H** Relative lipid ROS and MDA levels were measured in LoVo CSC streated with NALA (10 μM) or Ferr-1(1 μM) plus oxaliplatin (Oxa) for 12 h. **I** Western blotting was performed to evaluate the expression of GCLC, Pan Kla, H4K12la, and GPX4 in LoVo CSCs treated with 2-DG (10 μM) or Ferr-1(1 μM) plus oxaliplatin (Oxa). **J**, **K** Relative lipid ROS and MDA levels were measured in LoVo CSCs treated with 2-DG (10 μM) or Ferr-1(1 μM) plus oxaliplatin (Oxa) for 12 h. Three biological replicates were shown. The presented data show the mean ± SD. Comparisons were conducted using one-way ANOVA with Tukey’s test. *p < 0.05, **p < 0.01, ***p < 0.001.

To investigate the downstream targets of H4K12la in CCSCs chemoresistance, we performed Chromatin Immunoprecipitation followed by sequencing (ChIP-seq) using an anti-H4K12la antibody. ChIP-seq analysis showed that H4K12la was enriched in the promoter regions of specific genes (Fig. 6A and Supplementary Fig. S7A, B). KEGG pathway analysis revealed that H4K12la-specific genes were enriched in the cysteine and methionine metabolism, as well as in cancer-associated pathways (Fig. 6B). Abnormal cysteine metabolism is closely associated with ferroptosis [14, 27], so the above evidence indicates that histone lactylation may mediate tumor development, recurrence, and ferroptosis-related pathways. Next, the ChIP-seq data were combined with the RNA-seq data of the transcriptome of cells treated with oxaliplatin, either with or without lactate, and the set of genes associated with the ferroptosis pathway. This analysis identified GCLC as an anti-H4K12la-ChIP target gene, with significantly increased mRNA levels in lactate-treated cells during chemotherapy, and it was associated with the ferroptosis signaling pathway (Fig. 6C). Notably, analysis of the TCGA database revealed that GCLC is highly expressed in colorectal tumors (Supplementary Fig. S7C). To examine whether H4K12la activates GCLC transcription, we further analyzed the ChIP-seq data and found that H4K12la enrichment at GCLC promoter, and two peaks were identified (Fig. 6D), implying that H4K12la may transcriptionally activate GCLC. CUT&Tag-qPCR assays further confirmed H4K12la enrichment in the GCLC promoter regions, with enrichment levels increased by NALA treatment (Fig. 6E and Supplementary Fig. S7D). To determine whether H4K12la activates GCLC transcription, CCSCs were treated with NALA, resulting in a dose-dependent increase in GCLC mRNA and protein expression (Supplementary Fig. S7E, F). Furthermore, inhibition of H4K12la reduced GCLC expression at both the mRNA and protein levels (Supplementary Fig. S7G–L). Notably, analysis of the TCGA database revealed a positive correlation between GCLC expression and both EP300 (*R* = 0.303, *P* < 0.001) and LDHA (*R* = 0.266, *P* < 0.001) in CRC (Supplementary Fig. S7M, N). To test whether NALA prevents oxaliplatin-induced ferroptosis in CCSCs, we observed that NALA, similar to Ferr-1(a ferroptosis inhibitor) increased the protein expression of GCLC and GPX4, and reduced lipid ROS and MDA levels in CCSCs (Fig. 6F–H and Supplementary Fig. S8A–C). Importantly, treatment with 2-DG inhibited the protective effect of Ferr-1 against oxaliplatin-induced ferroptosis in CCSCs, leading to a reduction in GCLC and GPX4 protein expression, as evidenced by increased lipid ROS and MDA levels (Fig. 6I–K, and Supplementary Fig. S8D–F). Collectively, these findings show that H4K12la regulates GCLC transcription and plays a crucial role in inhibiting chemoresistance in CCSCs via ferroptosis.

### Inhibition of GCLC improves sensitivity to chemotherapy

**Fig. 7:**
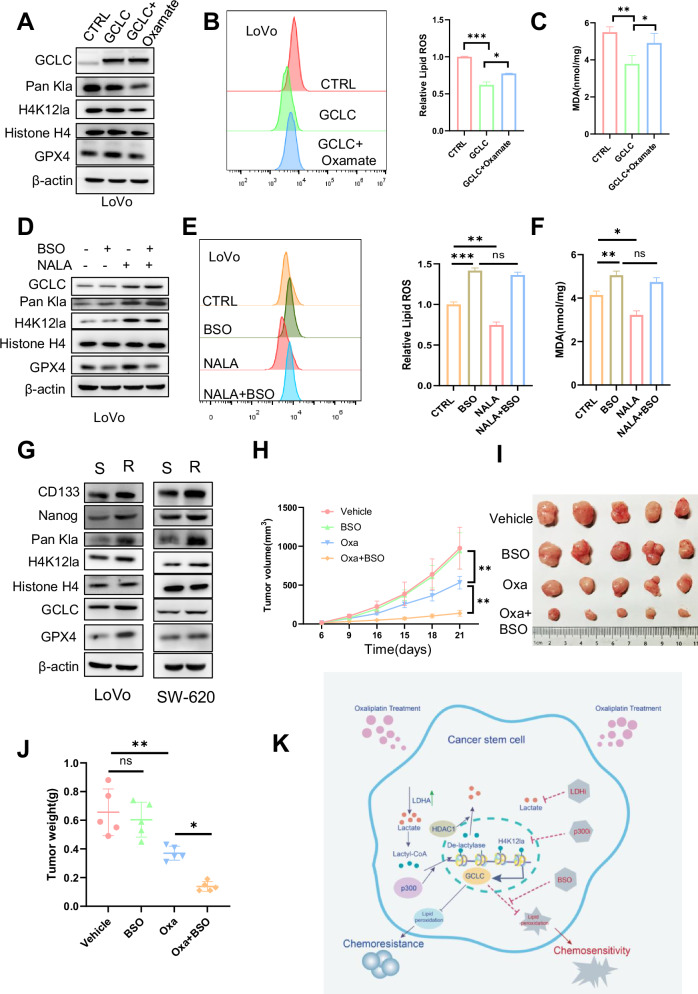
Inhibition of GCLC improves sensitivity to chemotherapy in CCSCs. **A** Western blot analysis was performed to test the expression of GCLC, Pan Kla, H4K12la, and GPX4 in LoVo CSCs overexpressing GCLC and treated with or without oxamate (10 mM). **B**, **C** Relative lipid ROS and MDA levels were measured in LoVo CSCs overexpressing GCLC, and treated with or without oxamate (10 mM). Cells were treated with oxaliplatin (5 μM) for 12 h to induce ferroptosis. **D** Western blot was performed to evaluate the expression of GCLC, Pan Kla, H4K12la, and GPX4 in LoVo CSCs treated with NALA and/or BSO. **E**, **F** Relative lipid ROS and MDA levels were measured in LoVo CSCs treated with oxaliplatin (5 μM) for 12 h. **G** Western blot analysis was performed to test the expression of GCLC, Pan-Kla, H4K12la, and GPX4 in LoVo and SW-620 CSCs; S, sensitive; R, resistant. **H**–**J** Nude mice were injected subcutaneously with LoVo CSCs and then received injections of oxaliplatin at 10 mg/kg twice a week, BSO at 300 mg/kg once a day; Tumor volumes were measured every 3 days (*n* = 5) and growth curves were plotted (**H**); Harvested tumors were photographed (**I**); and weights (**J**) were measured (*n* = 5). **K** Schematic representation of the proposed model. Three biological replicates were shown. Data are presented as mean ± standard deviation. Comparisons were conducted using one-way ANOVA with Tukey’s test, and statistical analyses were performed using Student’s t-test. *p < 0.05, **p < 0.01, ***p < 0.001, ns indicates no significant difference.

GCLC is a key enzyme in glutathione (GSH) synthesis, which prevents ferroptosis by inhibiting lipid peroxidation [18]. To determine whether GCLC function depends on H4K12la, our results showed that the expression of GPX4 and levels of lipid ROS and MDA in CCSCs transfected with GCLC were reversed by treatment with oxamate or 2-DG (Fig. 7A–C, and Supplementary Fig. S9A–I). To investigate whether NALA therapy could rescue ferroptosis caused by GCLC loss-of-function, CCSCs treated with the GCLC inhibitor BSO or GCLC knockdown did not show a significant increase in GPX4 expression, nor did lipid ROS and MDA levels change notably following NALA treatment (Fig. 7D–F and Supplementary Fig. S10A–I). Additionally, in vivo, tumorigenicity of NC and GCLC knockdown CCSCs was examined using limiting dilution assays (LDAs), monitoring tumor latency, incidence, and growth rate. Implantation of 100,000, 10,000, and 1000 shNC and GCLC knockdown CCSCs in nude mice. GCLC knockdown exhibited reduced tumor-initiating capacity and formed smaller tumors. These in vivo results demonstrated that GCLC suppression reduced the tumorigenic capacity of CCSCs (Supplementary Fig. S10J–L). These findings demonstrate that the inhibition of ferroptosis by histone lactylation in CCSCs is primarily mediated by GCLC.

Compared to oxaliplatin-sensitive parental cells, oxaliplatin-resistant cells exhibited significantly increased cell viability (Supplementary Fig. S11A, B). Western blot analysis showed that oxaliplatin-resistant CCSCs expressed higher levels of Pan Kla, H4K12la, CD133, Nanog, GCLC, and GPX4 (Fig. 7G). Lipid ROS and MDA levels were significantly lower in drug-resistant CCSCs (Supplementary Fig. S11C–F). These results imply that the ferroptosis signaling pathway may be mediated by in drug-resistance of CCSCs. Drug-resistant CCSCs were treated with BSO to investigate the possible therapeutic strategies. The results demonstrated that the viability of BSO-treated cells significantly decreased (Supplementary Fig. S11G–J). To further assess the role of GCLC in chemotherapy-resistant CCSCs in vivo, LoVo sphere cells were subcutaneously injected into nude mice. The results showed that the tumor growth rate in nude mice treated with oxaliplatin in combination with BSO was significantly inhibited (Fig. 7H–J). These data suggest that GCLC inhibition enhances the chemosensitivity of drug-resistant CCSCs. Taken together, targeting GCLC enhances the efficacy of chemotherapy against CCSCs.

## Discussion

Lactylation is a recently identified post-translational modification that facilitates tumor progression [5, 6, 8, 28, 29]. However, the molecular mechanism underlying the lactylation of CCSCs remains unclear. Our data show that lactylation levels are higher in CCSCs than in CCDCs. We found that H4K12 is lactylated by p300 and de-lactylated by HDAC1 in CCSCs. Interestingly, our results demonstrate that increased H4K12 lactylation in CCSCs promotes GCLC expression, which inhibits ferroptosis and leads to chemoresistance. Inhibitors of p300 and LDHA have been demonstrated to enhance chemosensitivity. Furthermore, GCLC inhibitors have been shown to significantly enhance the chemotherapy-mediated killing of CCSCs in vitro and in vivo (Fig. 7K).

Lactylation modifications are important PTMs that regulate a multitude of cellular processes [3, 30]. Elevated histone lactylation is associated with the oncogene expression and accelerates tumor progression [6, 31–33]. Here, we report for the first time that histone lactylation in CCSCs enhances GCLC expression, thereby promoting chemotherapy resistance. The investigation of lactylation, including non-histone lactylation, is of great significance. For example, lactylation of METTL16 at K229 results in cuproptosis in gastric cancer [34]. p53 lactylation at K120 and K139 contributes to cancer cell behavior [35]. Our experiments revealed that CCSCs exhibited a higher level of non-histone lactylation than differentiated cancer cells. Further research is essential to elucidate the function of non-histone lactylation in CCSCs.

The regulatory mechanisms of lysine lactylation, as mediated by lactyl-CoA writers, readers, and erasers, remain poorly understood. p300 is a transcriptional coactivator and lysine acetyltransferase that plays a critical role in tumorigenesis [36, 37]. A recent study showed that p300, a lactyltransferase, promotes histone lactylation in macrophages to regulate Arg1 expression, thereby facilitating wound healing [3]. Additionally, p300 has been identified as a writer of H3K18la, which regulates YTHDF2 expression, facilitating melanoma progression [6]. Our study showed that p300-mediated lactylation at H4K12 is crucial for the expression of ferroptosis-related genes. Other lactyltransferases have been recently identified that AARS1 mediates global lysine lactylation, thereby promoting tumor progression [35, 38]. Acetyltransferases such as CBP, KAT5, and KAT8 have also been demonstrated to function as lactyltransferases [5, 39, 40]. A previous study identified HDAC1-3 and SIRT1-3 as lysine delactylases in vitro, with HDAC1 and HDAC3 exhibiting site-specific delactylase activity in cells [24]. In our study, we identified HDAC1 as a delactylase of H4K12la in CCSCs. However, it is not yet known whether p300 and HDAC1 are involved in other histone lactylation modifications. These findings provide a theoretical foundation for the development of histone lactylation inhibitors.

Chemotherapy and radiotherapy resistance are intrinsic properties of CCSCs, with mechanisms including upregulation of drug efflux pumps, superior DNA repair capacity, and enhanced protection against ROS [41, 42]. One study demonstrated that H3K18la enhances resistance to bevacizumab in colorectal cancer by promoting RUBCNL expression [31]. Histone lactylation has also been linked to cisplatin resistance in bladder cancer cells [43]. Our results suggested that histone lactylation is a novel regulatory mechanism in drug resistance among CCSCs. Recent studies have suggested that targeting ferroptosis could provide novel therapeutic opportunities for tumors refractory to conventional treatments [44]. Previous studies have proposed that targeting SLC7A11 or GPX4 may enhance tumor cell sensitivity to chemotherapy and radiation by inducing ferroptosis [45, 46]. Therefore, targeting glutathione metabolism to induce ferroptosis represents a promising approach for colorectal cancer treatment. In this study, we found that histone lactylation in CCSCs activates the ferroptosis defense system by promoting GCLC expression. Further research on the role of epigenetics in ferroptosis is necessary to investigate whether other histone lactylation modifications affect the expression of ferroptosis-related genes.

Glutamate cysteine ligase (GCL) is the first rate-limiting enzyme in GSH biosynthesis, composed of catalytic GCLC and modifier GCLM subunits [47]. The GCLC subunit contains all the enzyme catalytic functions and can operate in vivo as a monomer, which is essential for GCL activity [48]. Several studies have demonstrated that the expression of GCLC is significantly elevated in tumor tissues and positively correlates with prognosis in patients with malignancies [20, 49]. Previous studies have demonstrated that reducing the expression of NRF2 and GCLC promotes tumorigenesis, growth, and enhanced drug resistance [50]. In another study, the histone methyltransferase G9a has been found to activate GCLC expression and enhance tumor drug resistance [51]. Here, we revealed that increased H4K12la in CCSCs elevates GCLC expression, thereby inhibiting ferroptosis and contributing to chemoresistance. Clinical trials have confirmed the safety of the GCLC inhibitor BSO in combination with chemotherapy for other tumor types [52, 53]. Our results indicate that BSO increased CCSCs sensitivity to oxaliplatin. These findings provide a compelling rationale for targeting GCLC to improve therapeutic outcomes in CRC patients.

Collectively, our study provides preliminary evidence that histone lactylation in CCSCs activates the ferroptosis defense system, promoting chemoresistance through the upregulation of GCLC expression. Notably, treatment with p300i, LDHi, and BSO decreased chemoresistance, offering new insights into strategies for overcoming chemoresistance in cancer, particularly in CRC.

## Methods

### Cell lines and cell culturing

The colorectal cancer cell lines LoVo, SW-620, HCT-116, and the human embryonic kidney cell line HEK293T were obtained from the American Type Culture Collection (ATCC). LoVo, SW-620, and HEK293T cells were cultured in DMEM (12800; Gibco). FBS (10%, v/v; Gibco) and 1% penicillin/streptomycin (15140148; Gibco) were added to the DMEM. All cells were cultured at 37 °C in a 5% (v/v) CO2 incubator.

For CRC sphere cells, 1 × 10^5^ tumor cells were cultured on 6-well ultra-low adhesion culture plates (3471; Corning) with 2 mL of stem cell medium (DMEM/F12) (31330095; Gibco) with 1:50 B27(17504044; Gibco), 20 ng/mL FGF (450-33; PERROTECH), 20 ng/mL EGF (100-47; PERROTECH), 4 μg/mL heparin (CC101; Macgene), 100 μg/mL apo-transferrin (CC109; Macgene), and 1% penicillin/streptomycin as previously described.

### Antibodies and chemicals

The following commercially available primary antibodies were used: Anti-CD133 (64326; Cell Signaling Technology); Anti-Nanog (8800; Cell Signaling Technology); Anti-OCT4A (83932; Cell Signaling Technology); Anti-SOX2 (23064; Cell Signaling Technology); Anti-p300 (86377; Cell Signaling Technology); Anti-CBP (A25323; ABclonal); Anti-GCLC (A1038; ABclonal); Anti-GPX4(A214400; ABclonal); anti-LDHA (A0861; ABclonal); Anti-Pan Kla (PTM-1401; PTM BIO); Anti-histone H3 (PTM-6600; PTM BIO); Anti-histone H4(PTM-1015RM; PTM BIO); Anti-H4K12la(PTM-1411RM; PTM BIO); Anti-H4K5la(PTM-1407RM; PTM BIO); Anti-H4K16la(PTM-1417RM; PTM BIO); Anti-H4K8la(PTM-1415RM; PTM BIO); pan); Anti-Pan Ac(PTM-101; PTM BIO); The following secondary antibodies were used in the immunofluorescence assays: DyLight 549, Goat anti-rabbit IgG (A23320; Abbkine); DyLight 488, goat anti-mouse IgG (A23210; Abbkine); Goat anti-Mouse IgG-HRP (abs20001; absin); Goat anti-Rabbit IgG-HRP(abs20040; absin).

The reagents for RSL3 (HY-100218A), Erastin (HY-15763), Fer-1 (HY-100579), BSO (HY-106376), Lactate sodium (NALA) (HY-B2227B), Sodium oxamate (LDHi) (HY-W013032A), Trichostatin A(TSA) (HY-15144), 2-DG (HY-13966) were purchased from MedChemExpress (MCE). D-glucose (50–99–7), L-lactic acid (79–33–4), and oxaliplatin (61825-94-3) were purchased from Sigma-Aldrich. The p300 inhibitor (SGC-CBP30) (S7256) was purchased from Selleckchem.

### siRNAs and plasmids

The siRNAs targeting p300 were designed and synthesized using AUGCT (http://www.augct.com). The sequences are listed in Table S2. The expression vectors encoding pCMV-EP300 (human)-3×FLAG and pEnCMV-CREBBP (human)-HA were purchased from MiaoLing (www.miaolingbio.com). Additionally, the pcDNA3.1-HDAC1 (human) and pcDNA3.1-GCLC (human) -Flag plasmids were constructed by inserting the indicated DNAs -Flag into the indicated vector. The sequences are listed in Table S3.

### Establishment of stable cell lines

The lentiviral shRNA vector pLV3-U6-(shHDAC1 or shGCLC or shLDHA) with the MD2-G and PPAX three-pack system was used to obtain silencing-expression viruses. The sequences are listed in Table S2. The pLV3-CMV-HDAC1(human)-EGFP, MD2-G, and PPAX three-pack system were used to generate a high-expression virus. All viruses were transfected into the indicated cells using lipofection. After 12 h, the medium was replaced with fresh complete medium. After 48 h, stable cell lines were obtained using 1 μg/mL puromycin.

### Collection of cancer stem cells

Cultured CCSCs were harvested, stained with PE anti-human CD133 for 15 min at 4 °C, and then sorted using a BD FACS Aria II flow cytometer to separate CD133^+^ and CD133^-^ cells for further experiments.

### Cell viability assay

The viability of cells was evaluated using a Cell Counting Kit-8 (CCK-8; HY-K0301; MedChemExpress). LoVo and SW-620 cells were seeded in ultra-low adhesion 96-well plates at a density of 1 × 10^4^ cells per well and incubated for 24 h before treatment with varying concentrations of the test compounds. At the specified time points, 10 μL of CCK-8 solution was added to each well, and after 2 h of incubation, the absorbance of the plates was measured at 450 nm. Cell viability was calculated as a percentage of the negative control under the indicated conditions.

### Measurement of Lipid ROS Level

Lipid ROS levels were measured in each group of cells using flow cytometry. The experiment was conducted by inoculating the indicated cell lines in ultra-low adhesion six-well plates at a density of 5 × 10^5^ cells per well. The cells were cultured for 24 h and subsequently treated with the experimental compounds for the indicated times. The cells were collected, and incubated with PBS containing C11-BODIPY 581/591 (RM02821; ABclonal) for 30 min at 37 °C. Subsequently, the cells were resuspended in 500 μL of PBS and analyzed for lipid ROS levels using flow cytometry (FACSuite; BD Biosciences). Data from the FITC channels were collected for this purpose, and the mean fluorescence was analyzed using FlowJo Version 10.8 software.

### MDA assay

The collected cells were lysed, and the MDA content in the supernatant was measured using a Lipid Peroxide MDA Assay Kit (S0131S; Beyotime) according with the manufacturer’s instructions.

### Measurement of lactate level

For suspension culture, cell precipitates were collected by centrifugation and were washed twice with PBS. After adding an appropriate volume of PBS, the cells were disrupted using an ultrasonic cell crusher under ice-cold conditions. Finally, the Lactic Acid Assay Kit (A019-2-1; Nanjing Jiancheng) was used according to the manufacturer’s instructions.

### Immunofluorescence

The cells were seeded at a density of 5 × 10^4^ cells/well on 24-well coverslips. After treatment for the indicated time periods, the cells were fixed in 4% paraformaldehyde for 15 min at room temperature. Subsequently, the fixed cells were permeabilized in 0.1% Triton X-100 for 5 min and incubated with the primary antibody overnight at 4 °C. The cells were then washed three times with PBS and incubated with the secondary antibody for 1 h. Finally, DAPI was added and the cells were incubated for 15 min at room temperature. The cells were visualized using a fluorescence microscope and the resulting images were analyzed using the ImageJ software.

### Tissue microarray and immunohistochemistry (IHC)

A human colorectal cancer tissue microarray containing 90 primary lesions and adjacent normal colon tissues (Shanghai OUTDO Technology Co., Ltd, HColA80Su13) (Tables S6 and S7) was used to evaluate tissue lactylation. IHC analysis of the paraffin-embedded CRC specimens was performed following the manufacturer’s protocol. Two independent gastrointestinal cancer pathologists assessed immunohistochemical staining results. The IHC scores were based on the extent and intensity of staining. The percentage of positively stained cells was scored on a scale of 0-4 as follows: 0 ( < 10%), 1 (10-25%), 2 (26-50%), 3 (51-75%), or 4 ( > 75%). Staining intensity was scored on a scale of 0 (negative), 1 (weak), 2 (intermediate), or 3 (strong). The IHC score was calculated by multiplying the percentage of positively stained cells by the staining intensity, which ranged from to 0 to 12. An IHC score >7 indicated high expression, while a score ≤7 indicated low expression.

### Western blotting

The cells were washed three times in ice-cooled PBS and then lysed with either NP-40 or RIPA buffer, which was supplemented with a protease inhibitor cocktail (HY-K0010; MedChemExpress), for 30 min at 4 °C. Protein concentration was measured using a Bicinchoninic Acid (BCA) Assay kit (23225; Thermo Fisher Scientific). Next, 5× protein sample buffer was added to the protein samples, and the samples were boiled at 95 °C for 10 min. The proteins were separated by electrophoresis on a 10% SDS-PAGE gel and were subsequently transferred onto a PVDF membrane. The membranes were then incubated for 2 h at room temperature with a 5% nonfat milk buffer. Next, the membranes were incubated overnight at 4 °C with the indicated antibodies, followed by HRP-conjugated secondary antibodies for 2 h at room temperature. Finally, the membranes were visualized using a chemiluminescence kit (34 096; Thermo Fisher Scientific).

For immunoprecipitation (IP), proteins were first extracted, and the supernatant was then centrifuged and incubated overnight at 4 °C with Protein A/G Magnetic Beads (HY-K0202; MCE) and primary antibodies. Eluted proteins were subsequently collected for further analysis.

### Quantitative real-time PCR (qPCR)

The cells were lysed using an RNA isolator (R401-01; Vazyme), and total mRNA was extracted following the manufacturer’s instructions. HiScript Q RT SuperMix for qPCR (R123-01; Vazyme) was used to reverse transcribe the mRNA according to the manufacturer’s instructions. Detection was performed on an ABI 7300 QuantStudio3 PCR system using the ChamQ SYBR qPCR Master Mix (Q341-02; Vazyme). The sequences are listed in Table S4.

### Mass spectrometry analysis

Pan lactylated proteins were enriched using the IP method with a pan anti-Kla antibody and separated by SDS-PAGE. The gel bands of interest were sent to the National Institute of Protein Science, School of Life Sciences, Tsinghua University for testing.

### Animal experiments

All animal studies were performed under the guidelines and protocols approved by the Institutional Animal Care and Use Committee of Tongji Medical College, Huazhong University of Science and Technology (TJH-201901005). The experimental animal model consisted of female, 6-week-old nude mice. After random grouping (*n* = 5), the collected colorectal cancer stem cells were washed twice with PBS and mixed with Matrigel in a 1:1 ratio. Subsequently, 5 × 10^6^/ml cells were injected subcutaneously. In some experiments, mice were intraperitoneally injected with oxaliplatin (10 mg/kg) twice a week. The mice were intraperitoneally injected with sodium lactate (NALA) (120 mg/kg) once daily and administered LDHi (1000 mg/kg) every two days. p300i (15 mg/kg) was intraperitoneally administered every two days. BSO (300 mg/kg) was intraperitoneally administered once daily. Tumor size was quantified at three-day intervals. The mice were sacrificed three weeks after tumor implantation to excise the tumors, and xenografts were subsequently subjected to further analysis. For Limiting Dilution Analysis (LDA), the frequency of tumor-initiating cells and statistical significance were examined using the Extreme Limiting Dilution Analysis software (bioinf.wehi.edu.au/software/elda/index.html).

### Patient and information details

This study was approved by the Ethics Committee of Tongji Hospital (TJ-IRB20230934) approved this study. The clinical specimens were obtained from the Department of Gastrointestinal Surgery at the Tongji Hospital. Informed consent was obtained from all patients. Table S5 lists demographic information, such as age and sex.

### RNA sequencing

The cells were subjected to RNA extraction using TRIzol reagent following the manufacturer’s instructions. Subsequently, mRNA libraries were generated using Illumina strantotal RNA Prep for paired-end RNA sequencing. Finally, libraries were sequenced using the Illumina NovaSeq 6000 sequencer.

### Chromatin immunoprecipitation (ChIP)

DNA libraries were prepared according to the manufacturer’s instructions using the SimpleChIP Enzymatic Chromatin IP Kit (9003; Cell Signaling Technology) and the DNA Library Prep Kit for Illumina Systems (56795; Cell Signaling Technology). Next-generation sequencing was performed on an Illumina Systems platform (PRJNA1154817).

### CUT&Tag assay

The CUT&Tag kit (TD904; Vazyme) was used according to the manufacturer’s instructions with the specific antibody H4K12la. DNA samples from CUT&Tag immunoprecipitations were analyzed by RT-qPCR and normalized to the inputs using isotype-matched IgG as a negative control. Primers specific to the GCLC promoter and other sites were designed based on the ChIP-seq data presented herein. The primes used are listed in Table S4.

### Data source and processing

The single-cell RNA sequencing (scRNA-seq) datasets GSE205506 and GSE245552, sourced from the Gene Expression Omnibus (GEO) database (https://www.ncbi.nlm.nih.gov/geo/), were included in this study. These datasets comprise 26 untreated adult primary tumor samples. Lactylation-related genes have not been fully identified. Accordingly, we selected genes involved in lactate metabolism and glycolysis and integrated them with the findings of previously published studies on lactylation in order to identify genes related to lactylation. In total, 231 lactylation-related genes were identified.

### scRNA-seq analysis

The scRNA-seq data were subjected to screening and subsequent analysis using the R package Seurat. The quality of the scRNA-seq data was initially evaluated. Subsequently, the R package “harmony” was employed to mitigate the batch effect between samples, and “ScaleData” was utilized to normalize the scRNA-seq data, which was then subjected to principal component analysis (PCA). The UMAP function was employed to reduce the dimensionality, and the FindAllMarkers function was used to identify differentially expressed genes (DEGs) in different clusters. Subsequently, cells were clustered at a resolution of 0.8, and the single R package was used in conjunction with manual adjustments for cell annotation. Finally, the GSEA Base R package was utilized to calculate the lactylation score for each cell based on lactylation-related genes.

### Statistical analysis

Statistical analyses were performed using GraphPad Prism version 10 (GraphPad Software, San Diego, CA, USA). The data is presented as mean ± SD. Survival curves were analyzed using Kaplan-Meier analysis and the log-rank test. Pearson^’^s correlation analysis and Spearman’s test were also performed. Student’s two-tailed t-test was used for comparisons between two groups and ANOVA with Tukey’s test was used for comparisons between more than two groups of continuous data.

## Supplementary information

Supplementary figures

original western blots

## Data Availability

All data needed to evaluate the conclusions in the paper are present in the paper and/or the Supplementary Materials.
