# Supplementary material for: Histone lactylation enhances GCLC expression and thus promotes chemoresistance of colorectal cancer stem cells through inhibiting ferroptosis
Source: Cell Death Dis. 2025 Mar 20;16(1):193. doi: 10.1038/s41419-025-07498-z (PMC11926133; doi:10.1038/s41419-025-07498-z)
Supplement: Supplementary file 1 — Supplementary figures [file 41419_2025_7498_MOESM1_ESM.pdf]

**Extended data**

**Supplementary Figure legend**

**Supplemental Figure 1. Lactylation mediates CRC tumor development, related to Figure 1.**

(**A**) Gene set enrichment analysis (GSEA) of TCGA-COAD dataset was performed to evaluate changes in glycolysis in CRC tumors and normal specimens. ES = 0.3875, NP=0.0316. (**B**) Immunohistochemical (IHC) staining was used to assess lactylation levels in a tissue microarray. (**C**) Western blotting was used to assess the expression of Pan Kla in tumor and normal specimens; T, tumor; N, normal. (**D**) The UMAP plot of clustered single-cell RNA-seq datasets demonstrates the existence of distinct CRC tumor subpopulations. The cells were clustered into eight different groups, with each cell cluster labeled and colored according to its subcellular type. (**E**) Western blot analysis was performed to assess the expression of CD133, Pan-Kla, Nanog, and OCT4 in CRC sphere and adherent cells. (**F**) Western blot was used to assess the expression of H4K12la in tumor and normal specimens; T, tumor; N, normal. (**G**) A schematic diagram illustrating sphere cell sorting. (**H**) Western blot analysis was performed to evaluate the expression of CD133, Pan Kla, Nanog and H4K12la in $CD133^-$ and $CD133^+$ cells.

**Supplemental Figure 2. Lactate regulates the histone lactylation and stemness of CCSCs**.

(**A**) Western blot analysis was performed to determine the expression of CD133, Nanog, Pan Kla, and H4K12la in SW-620 CSCs treated with lactate for 24 hours. (**B**) Western blot analysis was performed to evaluate the expression of CD133, Nanog, Pan Kla, and H4K12la in SW-620 CSCs treated with NALA for 24 hours. (**C**) Western blot analysis was performed to assess the expression of CD133, Nanog, Pan Kla, and H4K12la in

SW-620 CSCs cultured in regular medium containing varying concentrations of glucose for 24 hours; Right: Intracellular lactate levels were measured. (**D**) IF staining was performed to observe H4K12la (red) in LoVo cells treated with lactate for 24 hours. Scale bar: 100 μm. (**E**) HCT-116 CSCs were exposed to 2-DG for 24 hours. Left: Western blot analysis was used to assess the expression of CD133, Nanog, Pan Kla, and H4K12la; Right: Intracellular lactate levels were measured. (**F**) SW-620 CSCs were cultured under hypoxic conditions for different durations. Left: Western blot analysis was used to assess the expression of CD133, Nanog, Pan Kla, and H4K12la; Right: Intracellular lactate levels were measured. (**G**) The expression of LDHA was analyzed in the TCGA-COAD and TCGA-READ datasets (normal=51, tumor=647). (**H**) Kaplan-Meier overall survival analysis of LDHA expression in the public colorectal cancer dataset (GSE17536) (n=177, Cox regression analysis). $p < 0.001$.

**Supplemental Figure 3. p300 is an H4K12la lactylase and HDAC1 is an H4K12la delactylase.**

(**A**) Western blot analysis was used to evaluate the expression of p300, CD133, Pan-Kla, Nanog, Pan Ac, and H4K12la in siP300 SW-620 CSCs. (**B**) IF assay was performed to assess the expression of H4K12la and Pan Ac in siP300 SW-620 CSCs. Scale bar: 200 μm. (**C**) Western blot analysis was used to evaluate the expression of HDAC1, CD133, Pan-Kla, SOX2, Pan Ac, and H4K12la in shHDAC1 HCT-116 CSCs. (**D**) IF assay was performed to assess the expression of H4K12la and Pan Ac in shHDAC1 HCT-116 CSCs. Scale bar: 100 μm. (**E**) Western blot analysis was used to assess the expression of CD133, Pan Kla, Nanog, Pan Ac, and H4K12la in LoVo and SW-620 CSCs cultured in regular medium containing TSA for 24 hours. (**F**) Western blot analysis was used to assess the expression of CD133, Pan Kla, Nanog, Pan Ac, and

H4K12la in HCT-116 CSCs overexpressing HDAC1.

**Supplemental Figure 4. Inhibition of lactylation makes CCSCs responsive to chemotherapeutic agents, related to Figure 4.**

(**A**) Cell viability was assessed in SW-620 and LoVo CSCs treated with glucose and oxaliplatin for 24 hours. (**B**) Representative immunohistochemical images of H4K12la and Ki67 in tumor sections are shown. Scale bar, 50 μm. (**C**) Representative images of immunohistochemical staining for H4K12la and Ki67 in tumor sections. Scale bar, 50 μm. Three biological replicates were shown. The presented data shown represent the mean ± SD. Statistical analyses were performed using the Student's t-test. $^{*}p < 0.05$, $^{**}p < 0.01$.

**Supplemental Figure 5. Ferroptosis in CCSCs is inhibited by histones lactylation.**

(**A**) Cluster analysis of RNA expression levels in LoVo CSCs treated with oxaliplatin or oxaliplatin plus lactate. (**B**) KEGG enrichment analysis was conducted to identify the 20 most enriched KEGG terms, including ferroptosis. (**C** and **D**) Relative lipid ROS and MDA levels were tested in SW-620 CSCs treated with RSL3 (5 μM) or Erastin (5 μM) or oxaliplatin (5 μM) for 12 hours (n=3). (**E** and **F**) Relative levels of lipid ROS and MDA were measured in SW-620 CSCs treated with oxaliplatin (5 μM) and lactate for 12 hours (n=3). (**G** and **H**) Relative levels of lipid ROS and MDA were measured in LoVo CSCs treated with oxaliplatin (5 μM) and NALA for 12 hours (n=3). (**I** and **J**) Relative levels of lipid ROS and MDA were tested in SW-620 CSCs treated with oxaliplatin (5 μM) and NALA for 12 hours (n=3). (**K** and **L**) Relative levels of lipid ROS and MDA in were measured HCT-116 CSCs treated with oxaliplatin (5μM) and 2-DG for 12 hours (n=3). Three biological replicates were shown. The presented data show the mean ± SD; Comparisons were conducted using one-way ANOVA with

Tukey's test. *$p < 0.05$, **$p < 0.01$, ***$p < 0.001$. ns indicates no significant difference.

**Supplemental Figure 6. Ferroptosis is inhibited by histone lactylation in CCSCs.**

(**A** and **B**) Relative levels of lipid ROS and MDA were measured in shLDHA SW-620 CSCs treated with oxaliplatin (5 μM) for 12 hours (n=3). (**C** and **D**) Relative levels of lipid ROS and MDA were measured in siP300 SW-620 CSCs treated with oxaliplatin (5 μM) for 12 hours (n=3). (**E** and **F**) Relative levels of lipid ROS and MDA were measured in shHDAC1 HCT-116 CSCs treated with oxaliplatin (5 μM) for 12 hours (n=3). Three biological replicates were shown. The presented data show the mean ± SD; Comparisons were conducted using one-way ANOVA with Tukey's tes. *$p < 0.05$, **$p < 0.01$, ***$p < 0.001$.

**Supplemental Figure 7. Expression of GCLC is transcriptionally activated by histone lactation.**

(**A**) The H4K12la modification is primarily enriched at the TSS. (**B**) Genome-wide distribution of H4K12la- associated peaks in LoVo CSCs. (**C**) GCLC expression was analyzed in TCGA-COAD and TCGA-READ datasets: normal = 51, tumor = 647; ***p < 0.001.(**D**) CUT&Tag-qPCR assay was used to analyze H4K12la in the GCLC genomic region of SW-620 CSCs. (**E**) Quantitative real-time PCR (qPCR) analysis of GCLC mRNA levels in LoVo and SW-620 CSCs cultured in regular medium containing NALA for 24 hours (n=3). (**F**) Western blot analysis was used to assess GCLC expression in LoVo and SW-620 CSCs cultured in regular medium containing NALA for 24 hours. (**G**) qPCR analysis of GCLC mRNA levels in siP300 LoVo and SW-620 CSCs. (**H**) Western blot analysis was performed to assess GCLC expression in siP300 LoVo and SW-620 CSCs. (**I**) qPCR analysis of GCLC mRNA levels in shLDHA LoVo and SW-620 CSCs. (**J**) Western blot analysis was performed to assess GCLC expression

in shLDHA LoVo and SW-620 CSCs. (**K**) qPCR analysis of GCLC mRNA levels in LoVo and SW-620 CSCs cultured in regular medium containing oxamate for 24 hours. (**L**) Western blot analysis was used to assess GCLC expression in LoVo and SW-620 CSCs cultured in regular medium containing oxamate for 24 hours. (**M**) Gene expression correlation between GCLC and EP300 in TCGA-COAD and TCGA-READ datasets (n =647); R = 0.303, $p$ < 0.001. (**N**) Gene expression correlation between GCLC and LDHA in TCGA-COAD and TCGA-READ datasets (n=647); R = 0.266, $p$ < 0.001.

**Supplemental Figure 8**. **Histone lactylation defends against ferroptosis.**

(**A**) Western blot was performed to examine the expression of Pan Kla, H4K12la, GCLC, and GPX4 in SW-620 CSCs. The cells were treated with NALA (10 μM) or Ferr-1 (1 μM) along with oxaliplatin (5 μM). (**B** and **C**) Relative lipid ROS and MDA levels were tested in SW-620 CSCs treated with NALA (10 μM) or Ferr-1 (1 μM) along with oxaliplatin (5 μM) for 12 hours. (**D**) Western blot of Pan Kla, H4K12la, GCLC, and GPX4 in SW-620 CSCs treated with NALA (10 μM) or Ferr-1 (1 μM) along with oxaliplatin (5 μM). (**E** and **F**) Relative lipid ROS and MDA levels were evaluated in SW-620 CSCs treated with 2-DG (10 μM) and/or Ferr-1 (1 μM) along with oxaliplatin (5 μM) treatment for 12 hours. Three biological replicates were shown. The presented data show the mean ± SD. Comparisons were conducted using one-way ANOVA with Tukey's test. *$p$ < 0.05, **$p$ < 0.01, ***$p$ < 0.001. ns indicates no significant difference.

**Supplemental Figure 9. Inhibition of histone Lactylation suppresses GCLC function.**

(**A**) Western blot analysis was used to assess the expression of GCLC, Pan Kla, H4K12la, and GPX4 in HCT-116 CSCs overexpressing GCLC and treated with

oxamate (10 mM). (**B** and **C**) Relative lipid ROS and MDA levels were assayed in HCT-116 CSCs overexpressing GCLC and treated with oxamate (10 mM) and oxaliplatin (5 μM) for 12 hours. (**D**) Western blot analysis was used to assess the expression of GCLC, Pan Kla, H4K12la, and GPX4 in HCT-116 CSCs overexpressed GCLC and treated with 2-DG (10 μM). (**E** and **F**) Relative lipid ROS and MDA levels were examined in HCT-116 CSCs overexpressed GCLC and treated with 2-DG (10 μM) plus oxaliplatin (5 μM) for 12 hours. (**G**) Western blot analysis was used to assess the expression of GCLC, Pan Kla, H4K12la, and GPX4 in LoVo CSCs overexpressing GCLC treated with or without 2-DG (10 μM). (**H** and **I**) Relative lipid ROS and MDA levels were tested in LoVo CSCs overexpressing GCLC and treated with or without 2-DG (10 μM). Cells were treated with oxaliplatin (5 μM) for 12 hours to induce ferroptosis. Three biological replicates were shown. The data represent the mean ± SD. Comparisons were conducted using one-way ANOVA with Tukey's test. *$p < 0.05$, **$p < 0.01$, ***$p < 0.001$, ****$p < 0.0001$.

**Supplemental Figure 10. Histone lactylation inhibits ferroptosis through GCLC.**

(**A**) Western blot analysis was used to assess the expression of GCLC, Pan Kla, H4K12la, and GPX4 in HCT-116 CSCs treated with NALA (10 μM) or BSO (200 μM). (**B** and **C**) Relative lipid ROS and MDA levels were measured in HCT-116 CSCs treated with oxaliplatin (5 μM) for 12 hours. (**D**) Western blot analysis was used to assess the expression of GCLC, Pan Kla, H4K12la, and GPX4 in shGCLC LoVo CSCs. (**E** and **F**) Relative lipid ROS and MDA levels were measured in LoVo CSCs treated with oxaliplatin (5 μM) for 12 hours. (**G**) Western blot analysis was performed to assess the expression of GCLC, Pan Kla, H4K12la, and GPX4 in shGCLC HCT-116 CSCs. (**H** and **I**) Relative lipid ROS and MDA levels were measured in HCT-116 CSCs treated

with oxaliplatin (5 μM) for 12 hours. (**J**) Limiting dilution assay (LDA) was performed for sphere cells injected subcutaneously into nude mice to determine tumor-initiating frequency (TIF). TIF, tumor-initiating frequency. Tumors were harvested at 42 days post implantation and represented images were taken (**J**), (**K**)tumor weights and (**L**)tumor volumes were measured in mice injected with 10,000 cells. Three biological replicates were shown. The data shown represent the mean ± SD. Comparisons were conducted using one-way ANOVA with Tukey's test. *$p < 0.05$, **$p < 0.01$, ***$p < 0.001$, ns indicates no significant difference.

**Supplemental Figure 11. Enhanced chemosensitivity of drug-resistant CSCs by targeting GCLC.**

(**A and B**) Cell survival was evaluated in resistant and sensitive SW-620 and LoVo CSCs treated with oxaliplatin for 24 hours. (**C** and **D**) Relative lipid ROS and MDA levels were examined in SW-620 CSCs treated with oxaliplatin (5 μM) for 12 hours. (**E**) Relative lipid ROS and MDA levels were evaluated in SW-620 and LoVo CSCs treated with oxaliplatin (5 μM) for 12 hours. (**F**) Relative lipid ROS and MDA levels were tested in LoVo CSCs treated with oxaliplatin (5 μM) for 12 hours. (**G** and **H**) Cell survival was evaluated in the resistant LoVo and SW-620 CSCs treated with oxaliplatin and/or BSO for 24 hours. **(I** and **J)** Cell survival was evaluated in resistant LoVo and SW-620 CSCs treated with BSO and oxaliplatin for 24 hours. Three biological replicates were shown. The data shown represent the mean ± SD. Comparisons were conducted using two-way ANOVA with Tukey's test and Student's two-tailed t-test. **$p < 0.01$, ***$p < 0.001$, ****$p < 0.0001$.

Supplemental Fig.1

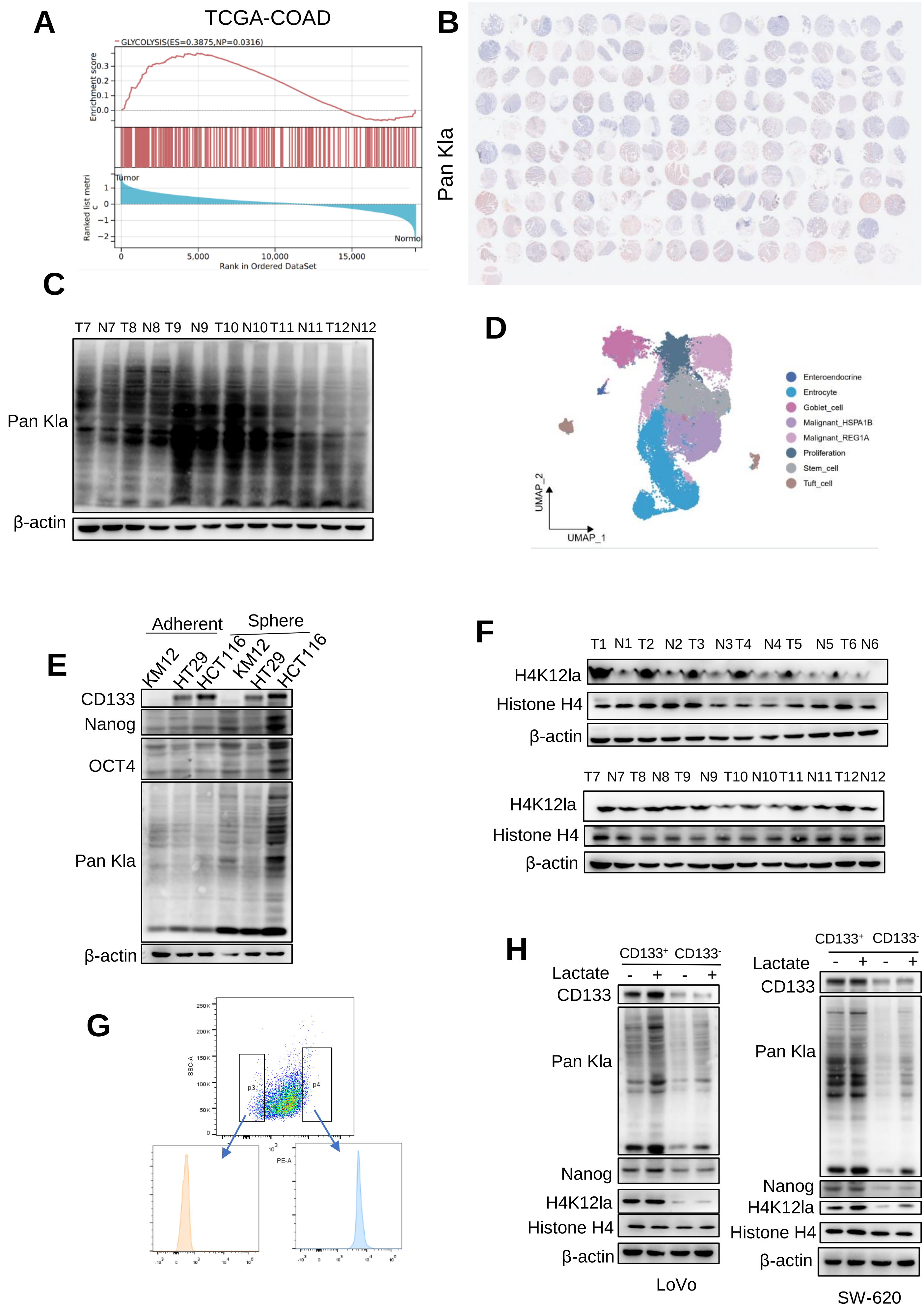

Supplemental Fig. 2

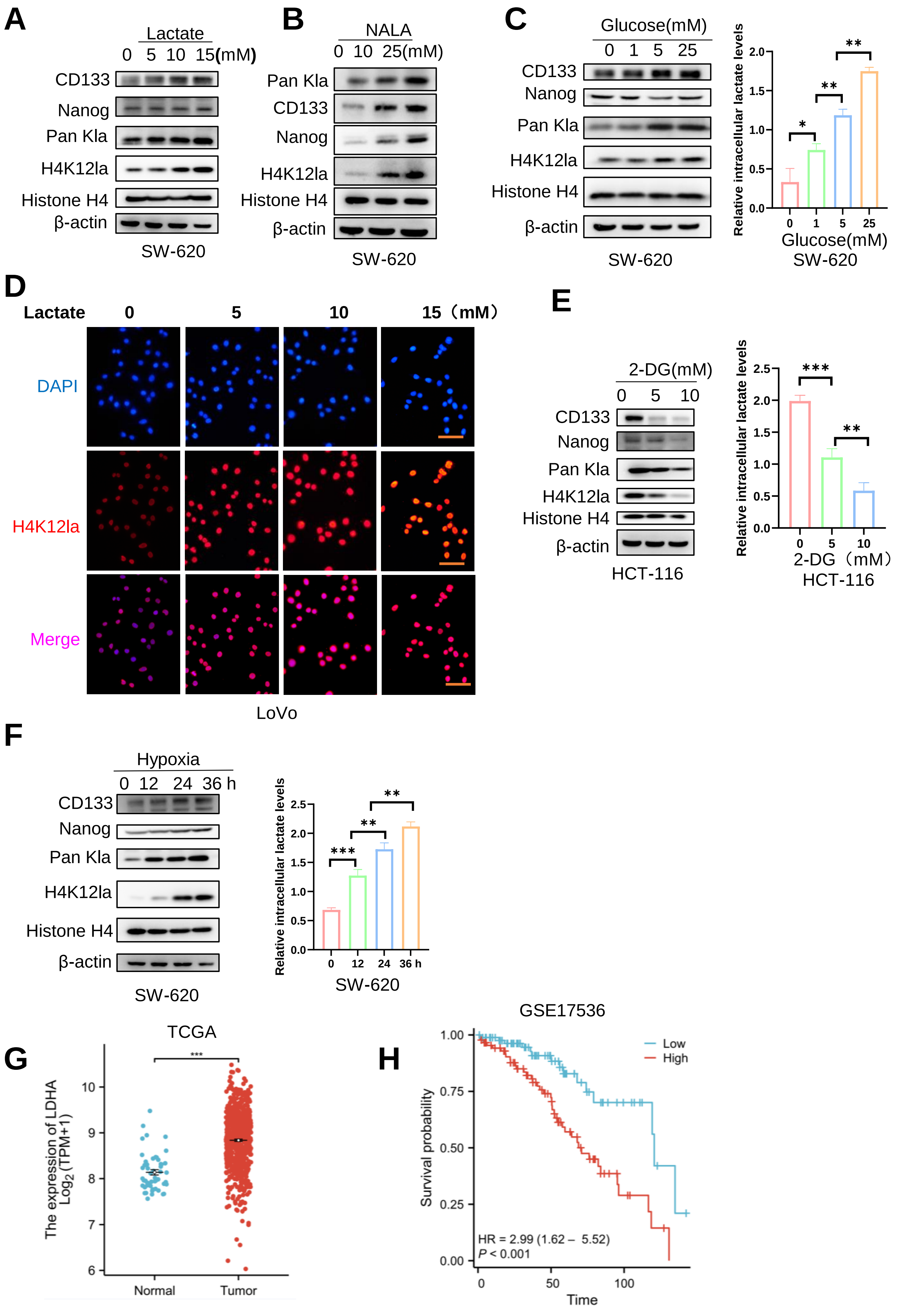

# Supplemental Fig. 3

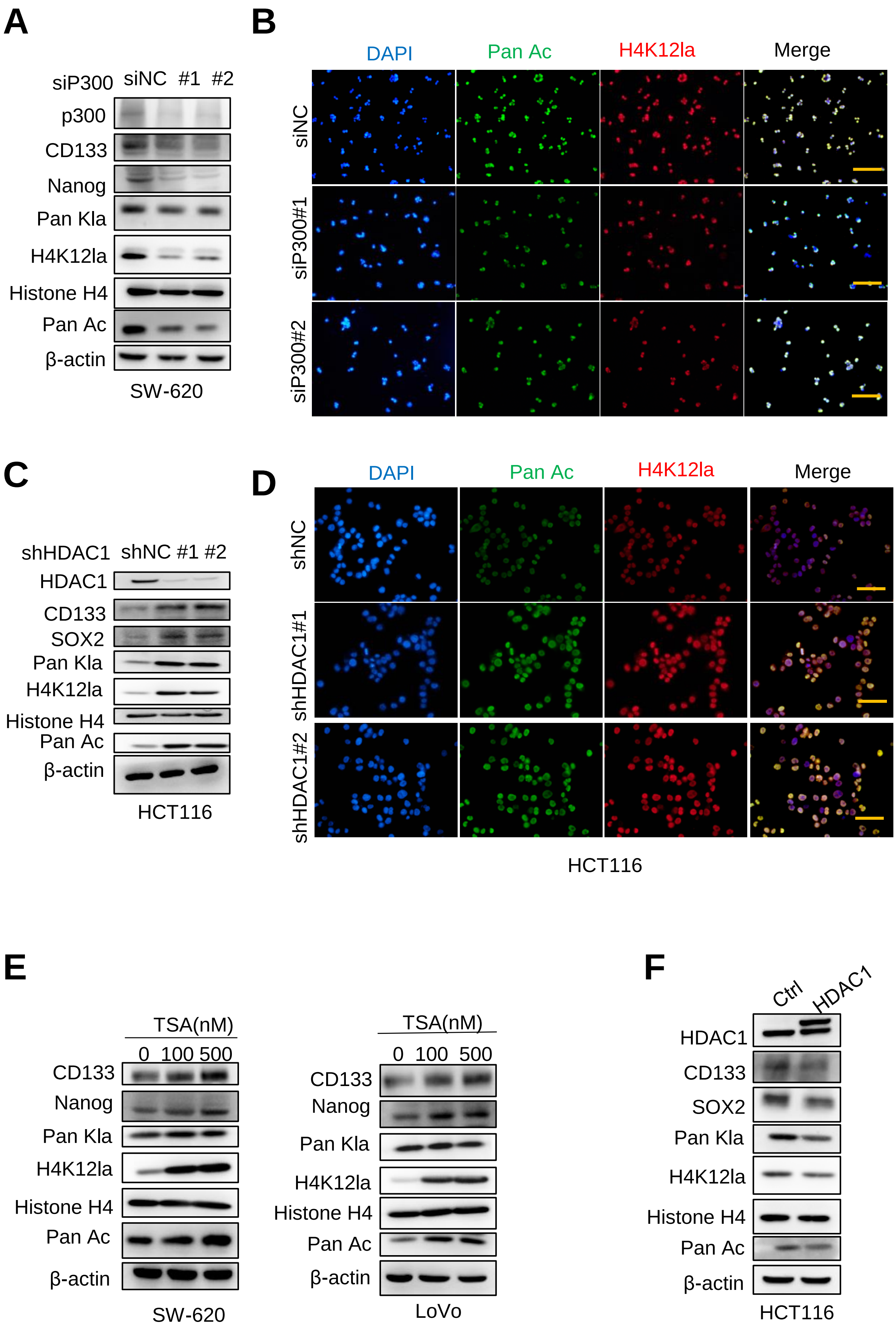

# Supplemental Fig. 4

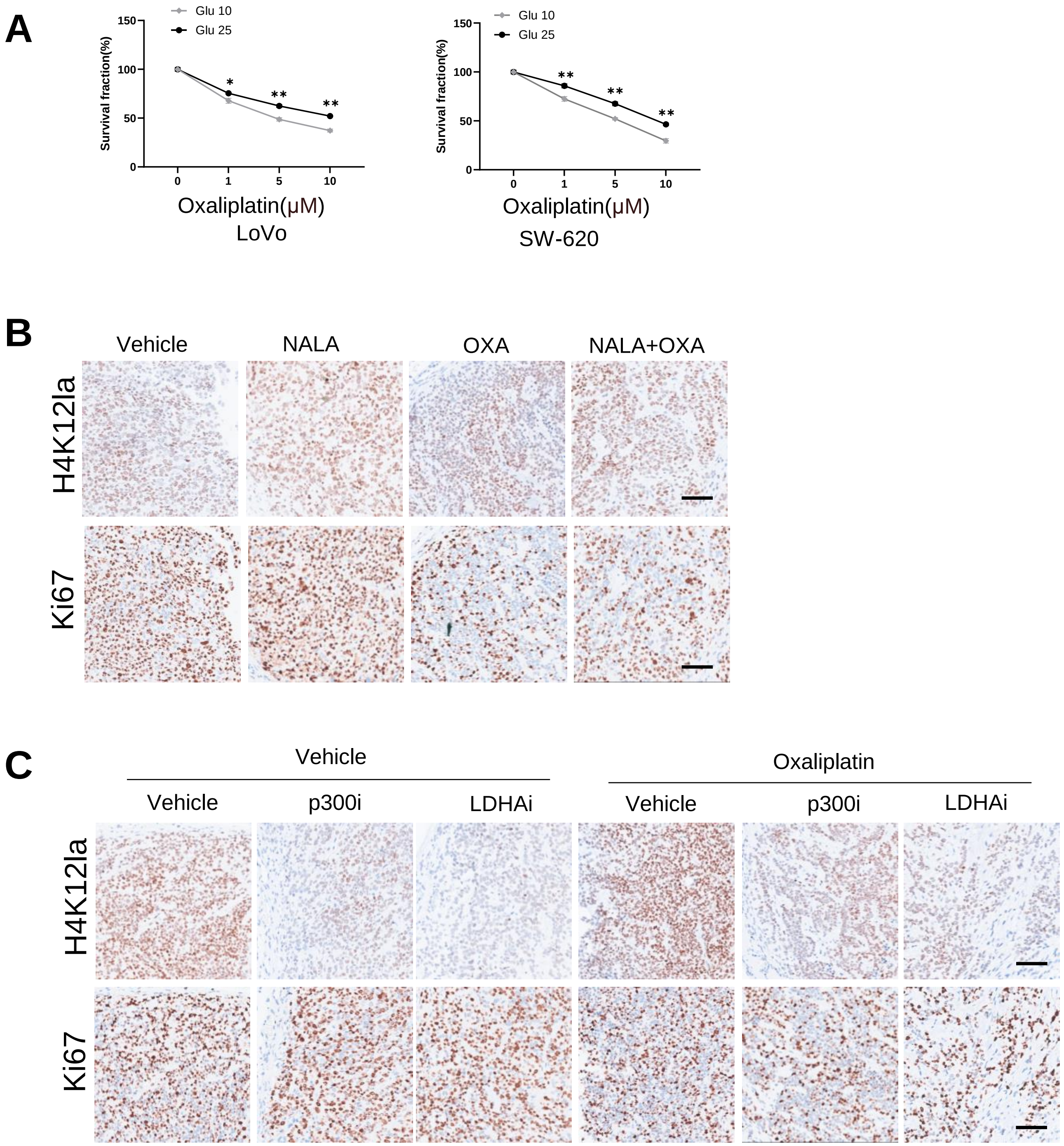

Supplemental Fig. 5

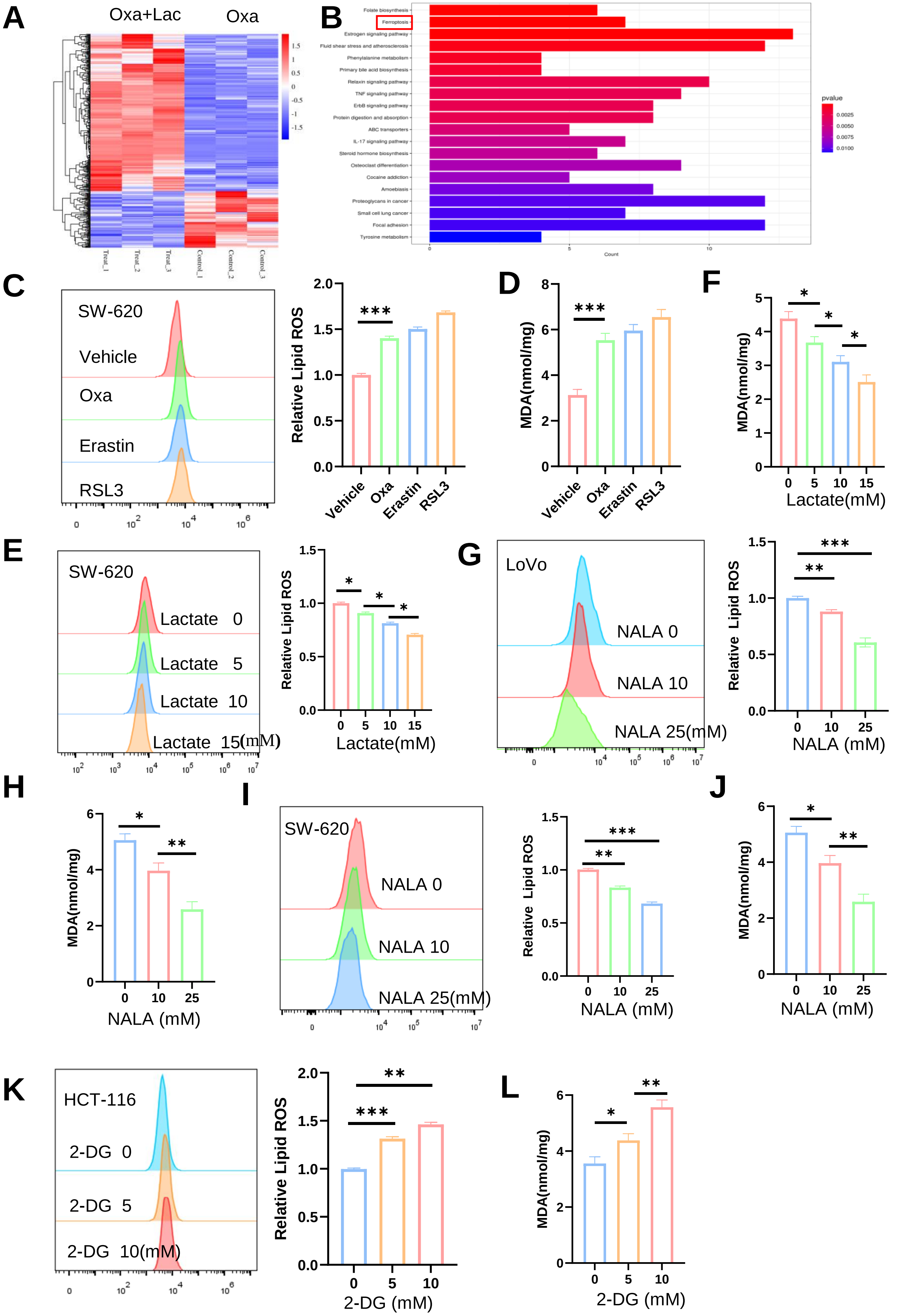

# Supplemental Fig. 6

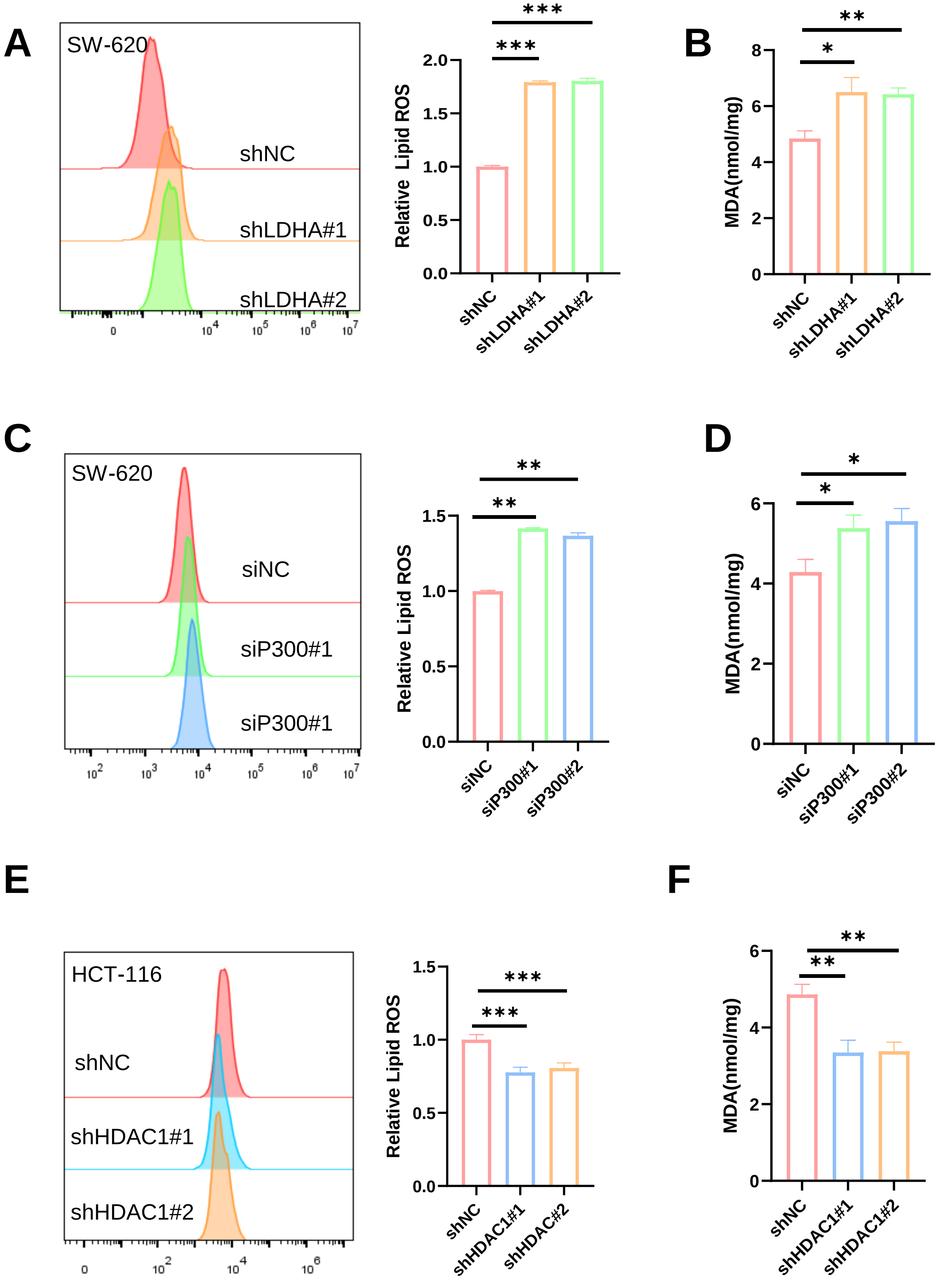

Supplemental Fig. 7

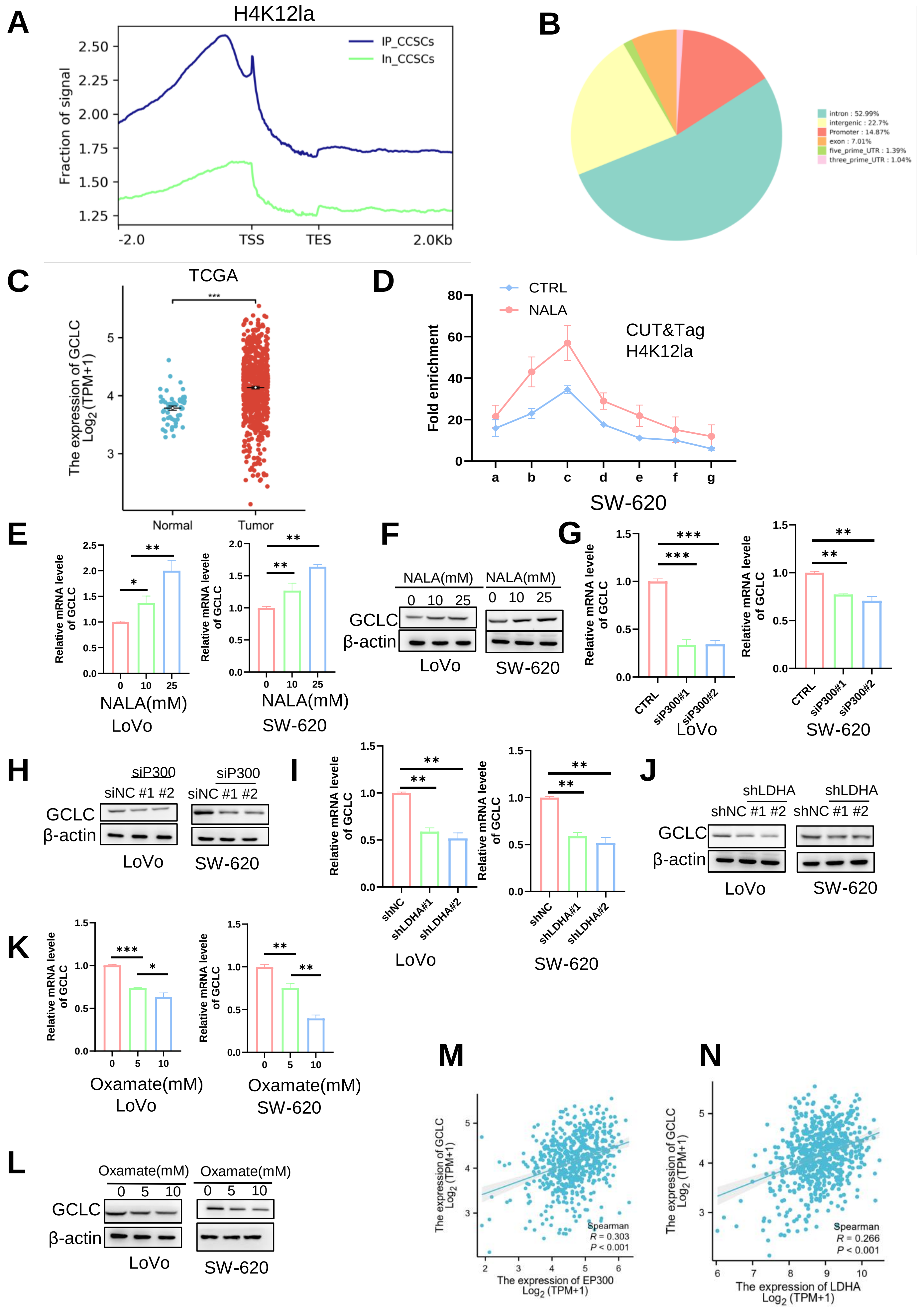

Supplemental Fig. 8

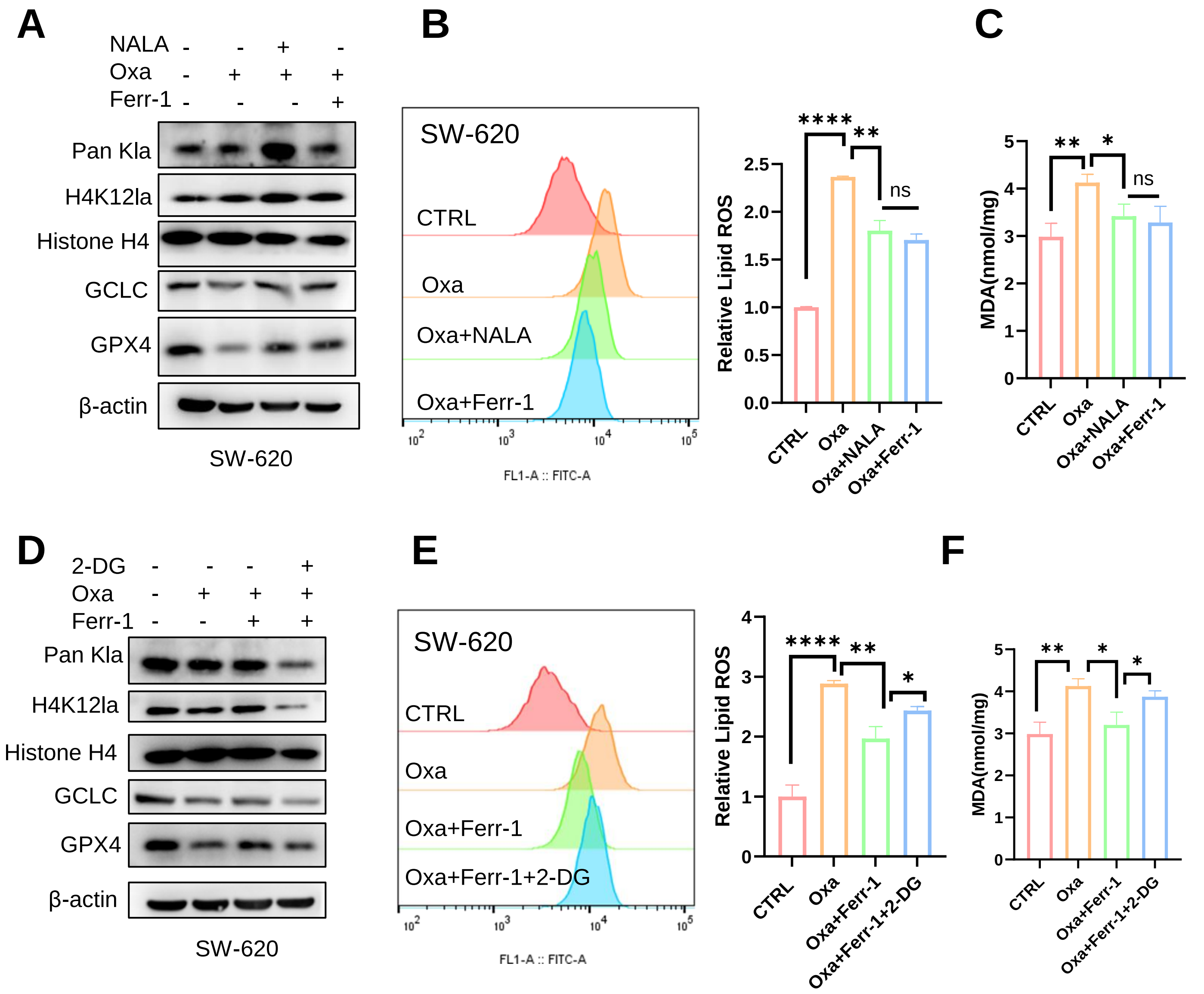

Supplemental Fig. 9

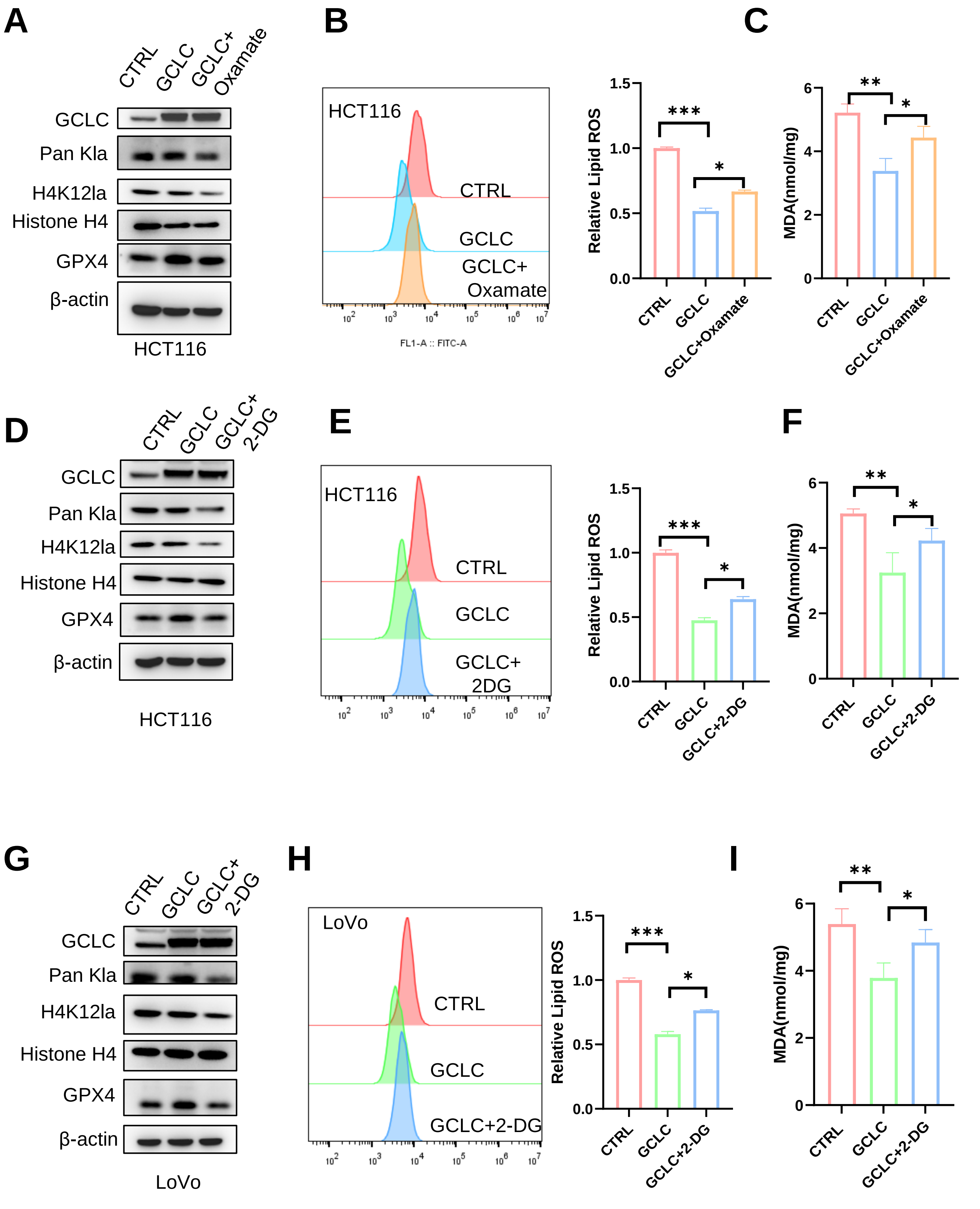

# Supplemental Fig. 10

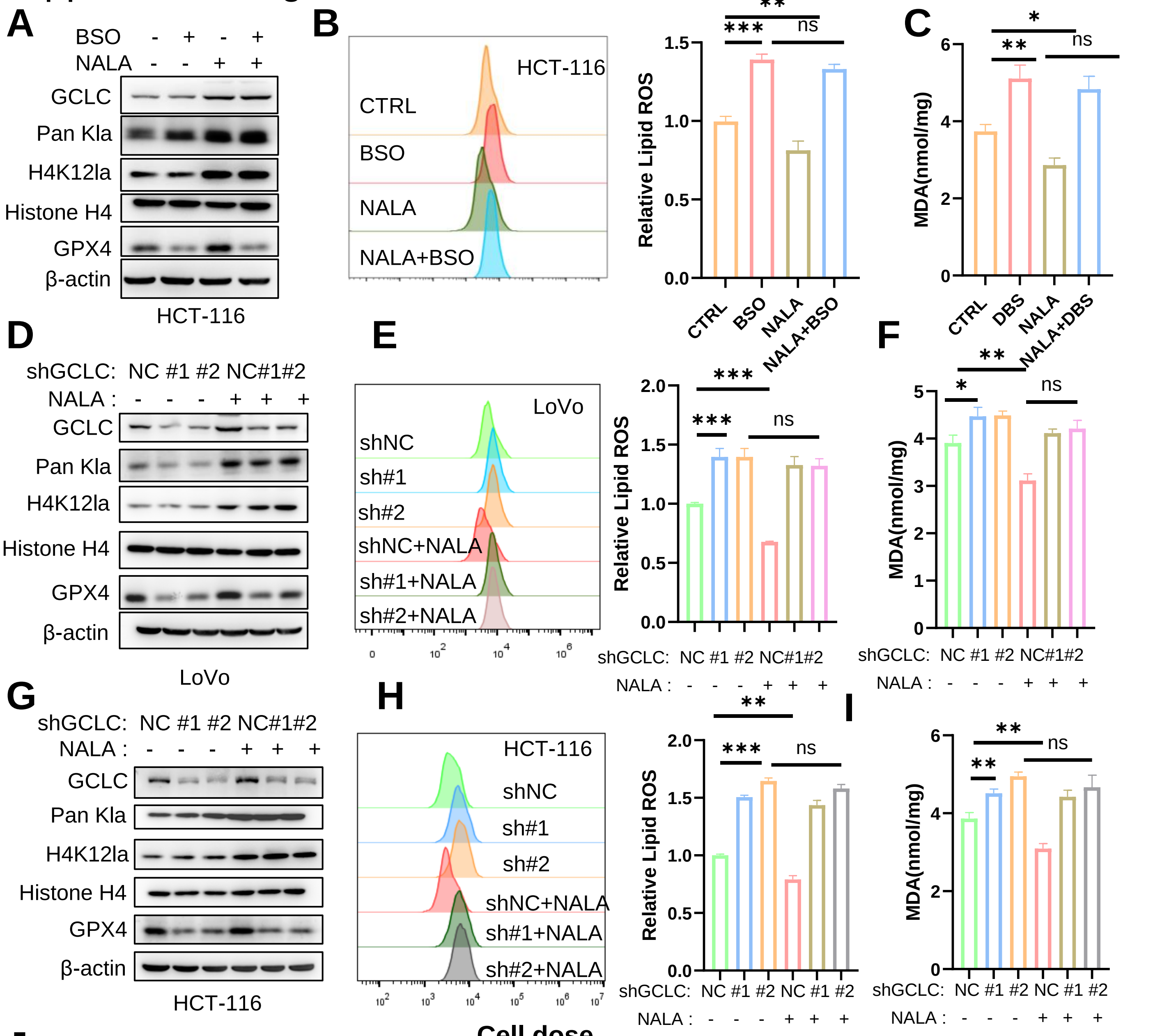


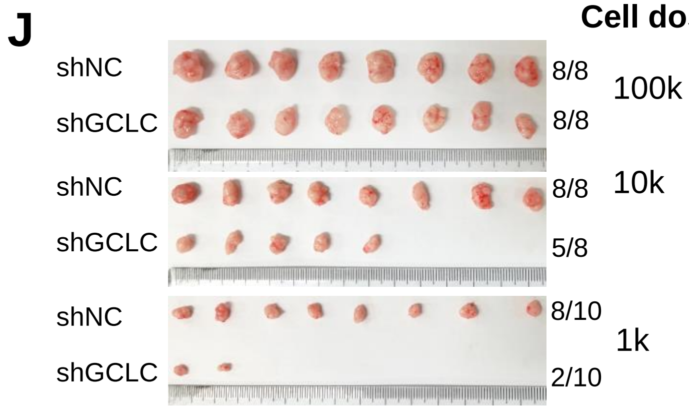


**Cell dose**

| Group | incidence 100k | 10k | 1k | TIF | *P* value |
|---|---|---|---|---|---|
| shNC | 8/8 | 8/8 | 8/10 | 1/621 (1/288-1/1342) | 5.11E+06 |
| shGCLC | 8/8 | 5/8 | 2/10 | 1/8451 (1/3814-1/18726) | |

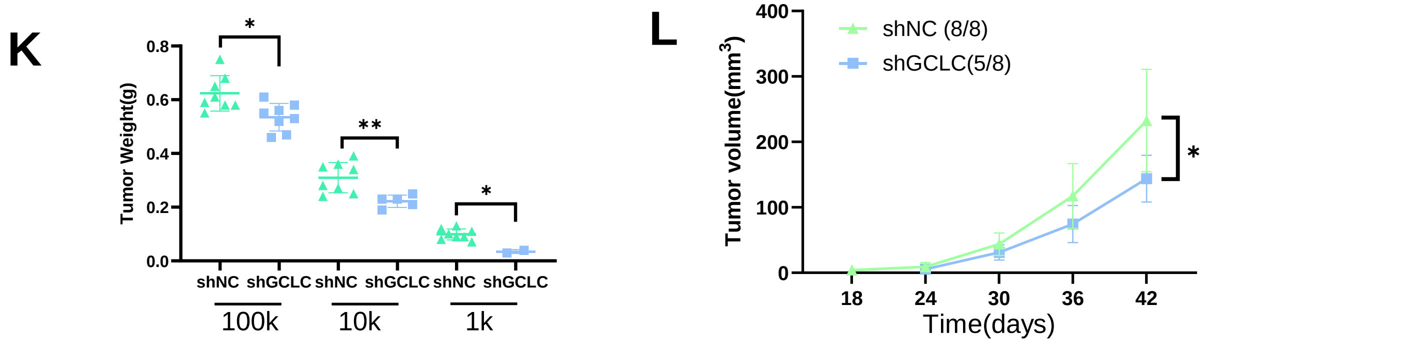

Supplemental Fig. 11

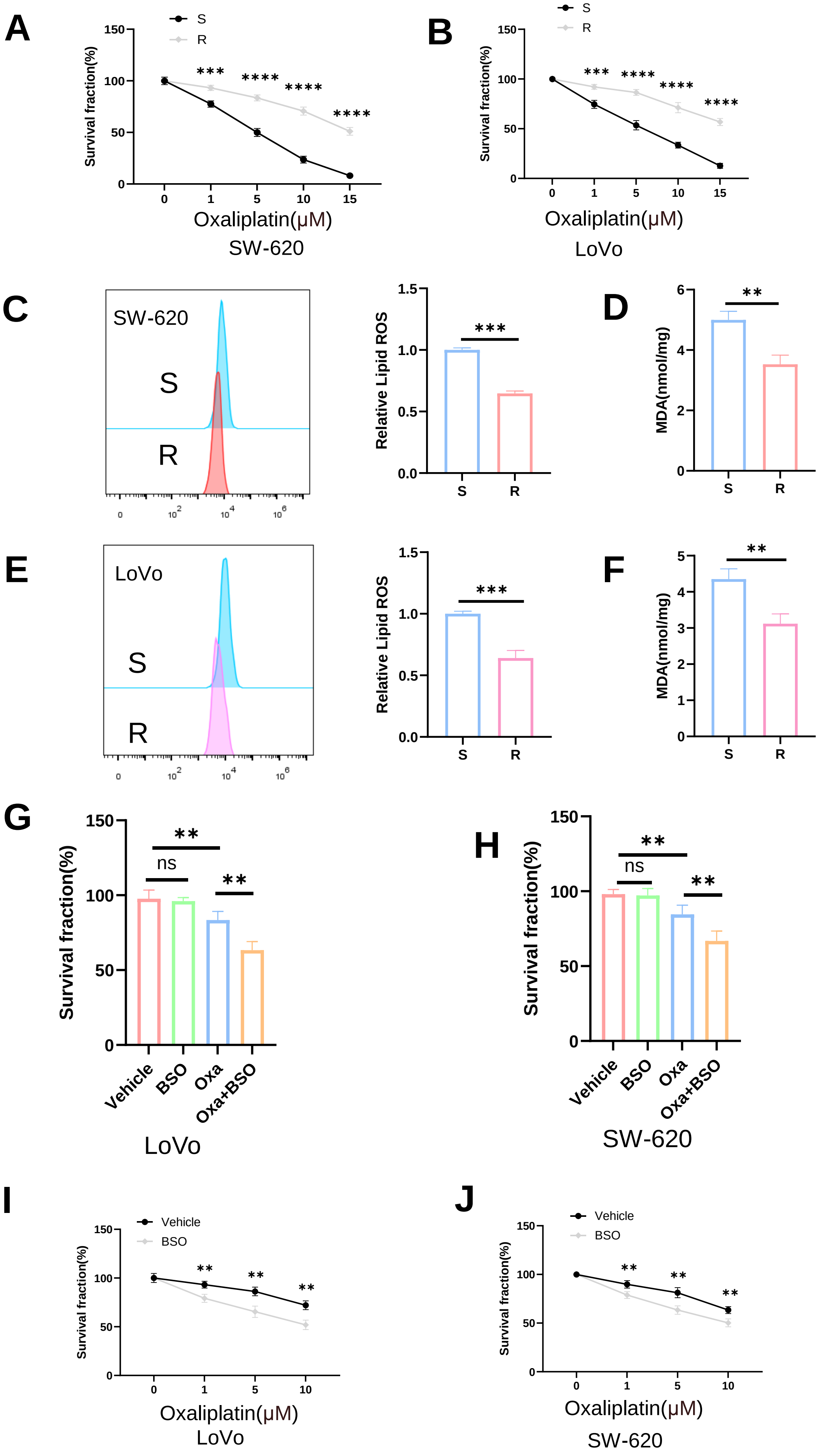

# Table S1 Pan Kla binding proteins

The file is included as an excel file

| Description | Score |
|---|---|
| Histone H4 | 63.54806781 |
| Histone H2B | 53.29506576 |
| Histone H2A | 50.1964041 |
| Histone H2A | 41.88940346 |
| Histone H1.2 | 28.27578676 |
| Histone H1.4 | 28.22905266 |
| Histone H2A | 25.06000197 |
| Histone H1.5 | 24.78438914 |
| Histone H1.10 | 15.30097985 |
| Histone H3.1 | 7.717903376 |
| Histone H3-7 | 5.093426466 |
| Histone H1.0 | 2.533623457 |
| CREBBP | 30.54 |
| EP300 | 7.63 |
| HDAC1 | 5.94 |

# Table S2 The sequence of siRNA and shRNA

| The sequence of siRNA and shRNA | |
|---|---|
| Gene | Sequence |
| sip300#1 | CAATTCCGAGACATCTTGAGA |
| sip300#2 | GCCTTCACAATTCCGAGACAT |
| sip300#3 | CCCGGTGAACTCTCCTATAAT |
| shLDHA#1 | CCACCATGATTAAGGGTCTTT |
| shLDHA#2 | CCAAAGATTGTCTCTGGCAAA |
| shLDHA#3 | CGTTTGAAGAAGAGTGCAGAT |
| shGCLC#1 | GCCATTGAAGAACAATAACTA |
| shGCLC#2 | CCAATTCTGAACTCTTACCTT |
| shGCLC#3 | CCGATGCAGTATTCTGAACTA |
| shHDAC1#1 | GCCGGTCATGTCCAAAGTAAT |
| shHDAC1#2 | CCGCAAGAACTCTTCCAACTT |
| shHDAC1#3 | GCTGCTCAACTATGGTCTCTA |

# Table S3 The prime used to construct the plasmids

| The prime used to construct the plasmids | | |
|---|---|---|
| Plasmid | Forward Prime | Reverse Prim |
| GCLC | ATGGGGCTGCTGTCCCAGGG | GTTGGATGAGTCAGTTTTACTTC |
| HDAC1 | ATGGCGCAGACGCAGGG | GGCCAACTTGACCTCCTCCTTGA |

# Table S4 The sequences of the primers for the qPCR

| The sequences of the primers for the qPCR | | |
|---|---|---|
| Gene | Forward Prime | Reverse Prime |
| GCLC | GGAGGAAACCAAGCGCCAT | CTTGACGGCGTGGTAGATGT |
| ACTB | CATGTACGTTGCTATCCAGGC | CTCCTTAATGTCACGCACGAT |
| GCLC a sit | TCTGGGGGAGGCTTCTTAGG | CCCGAAACCCATCGTGTCTG |
| GCLC b sit | GGGTGATTGGGTCGCAGTT | TTGCGTAAAGCGAGGCCGA |
| GCLC c sit | CGGGCGCTCACCTCATC | TGCACATCTACCACGCCG |
| GCLC d sit | CCATGGAAAGACACAAGCAAATCC | GGAACCTTCCCTTTCCTGTTTA |
| GCLC e sit | TTCACGAGGCTCACCTCATT | GGGTAGGCTTGTGCATTGGAT |
| GCLC f sit | TATCTGCGCAGCCTGTTTCT | GCCTAGTGGCTCAGATGCAA |
| GCLC g sit | GGCAGGGAAACGTGAAGTCTG | AAGTGGCACTACTGACTGATCCT |

# Table S5 The information of patients

| The information of patients | | | |
|---|---|---|---|
| ID | Gender | Age | TNM stage |
| 1010303147 | Female | 52 | T3N0M0 |
| 60010144349 | Female | 57 | T3N0M0 |
| 1009900018 | male | 25 | T4bN0M0 |
| 60010215306 | male | 64 | T3N0M0 |
| 1010428877 | Female | 64 | T2N0M0 |
| 1009949519 | Female | 56 | T3N0M0 |
| 1010442781 | male | 58 | T2N0M0 |
| 1010535308 | male | 48 | T3N0M0 |
| 1010485641 | male | 37 | T3N2M0 |
| 1010376570 | Female | 68 | T1N1M0 |
| 60010312945 | Female | 52 | T2N1M0 |
| 1010477088 | male | 51 | T3N0M0 |

# Table S6 colorectal cancer tissue microarray information.

| Sit location | Tissue Types | Score | Level | State of survival | survival time(month) | T | N | M | AJCC 7th edition clinical staging |
|---|---|---|---|---|---|---|---|---|---|
| A01 | Tumor | 5 | Low | 1 | 78 | T3 | N0 | M0 | II |
| A03 | Tumor | 1.5 | Low | 0 | 1 | T2 | N1b | M0 | III |
| A05 | Tumor | 7 | Low | 0 | 13 | T3 | N0 | M0 | II |
| A07 | Tumor | 6 | Low | 1 | 78 | T3 | N0 | M0 | II |
| A09 | Tumor | 7 | Low | 1 | 77 | T3 | N2b | M0 | III |
| A11 | Tumor | 7 | High | 0 | 7 | T4a | N2b | M0 | III |
| A13 | Tumor | 2 | Low | 0 | 22 | T3 | N0 | M1b | IV |
| A15 | Tumor | 2 | Low | 1 | 77 | T3 | N0 | M0 | II |
| A17 | Tumor | 2.5 | Low | 1 | 77 | T4a | N0 | M0 | II |
| B01 | Tumor | 4 | Low | 1 | 77 | T3 | N0 | M0 | II |
| B03 | Tumor | 7 | Low | 1 | 77 | T3 | N0 | M0 | II |
| B05 | Tumor | 12 | High | 0 | 44 | T4a | N0 | M0 | II |
| B07 | Tumor | 4 | Low | 1 | 77 | T2 | N0 | M0 | I |
| B09 | Tumor | 6 | Low | 1 | 76 | T3 | N0 | M0 | II |
| B11 | Tumor | 8 | High | 0 | 38 | T4a | N0 | M0 | II |
| B13 | Tumor | 8 | High | 0 | 13 | T4b | N1b | M0 | III |
| B15 | Tumor | 8 | High | 0 | 8 | T4a | N0 | M0 | II |
| B17 | Tumor | 4 | Low | 1 | 76 | T1 | N0 | M0 | I |
| C01 | Tumor | 12 | High | 0 | 56 | T4a | N2a | M0 | III |
| C03 | Tumor | 8 | High | 0 | 17 | T4a | N0 | M1b | IV |
| C05 | Tumor | 12 | High | 1 | 76 | T3 | N0 | M0 | II |
| C07 | Tumor | 12 | High | 0 | 12 | T3 | N0 | M0 | II |
| C09 | Tumor | 5 | Low | 1 | 76 | T2 | N0 | M0 | I |
| C11 | Tumor | 8 | High | 0 | 40 | T2 | N0 | M0 | I |
| C13 | Tumor | 7 | Low | 0 | 42 | T3 | N1a | M0 | III |
| C15 | Tumor | null | | 1 | 76 | T3 | N1b | M0 | III |
| C17 | Tumor | 2.5 | Low | 0 | 33 | T3 | N0 | M0 | II |
| D01 | Tumor | 5 | Low | 1 | 75 | T3 | N1b | M0 | III |
| D03 | Tumor | 12 | High | 1 | 75 | T3 | N0 | M0 | II |
| D05 | Tumor | 9 | High | 0 | 40 | T4a | N0 | M0 | II |
| D07 | Tumor | 5 | Low | 1 | 75 | T3 | N0 | M0 | II |
| D09 | Tumor | 5 | Low | 1 | 75 | T2 | N0 | M0 | I |
| D11 | Tumor | 8 | High | 0 | 23 | T4b | N1b | M0 | III |
| D13 | Tumor | 7 | Low | 1 | 75 | T3 | N0 | M0 | II |
| D15 | Tumor | 8 | High | 1 | 75 | T3 | N0 | M0 | II |
| D17 | Tumor | 6 | Low | 1 | 75 | T3 | N0 | M0 | II |
| E01 | Tumor | null | | 1 | 75 | T3 | N0 | M0 | II |
| E03 | Tumor | 6 | High | 1 | 75 | T3 | N0 | M0 | II |
| E05 | Tumor | 2.5 | Low | 1 | 75 | T3 | N0 | M0 | II |
| E07 | Tumor | 1.5 | Low | 1 | 75 | T3 | N0 | M0 | II |
| E09 | Tumor | 5 | Low | 0 | 13 | T4a | N1b | M0 | III |
| E11 | Tumor | 6 | Low | 1 | 74 | T3 | N0 | M0 | II |
| E13 | Tumor | 7 | Low | 0 | 39 | T4a | N1b | M0 | III |
| E15 | Tumor | 2 | Low | 1 | 74 | T2 | N0 | M0 | I |
| E17 | Tumor | 6 | Low | 0 | 13 | T3 | N0 | M0 | II |
| F01 | Tumor | 1.5 | Low | 0 | 1 | T3 | N0 | M0 | II |
| F03 | Tumor | 7 | Low | 1 | 73 | T3 | N0 | M0 | II |
| F05 | Tumor | 8.5 | High | 1 | 73 | T4a | N1b | M0 | III |
| F07 | Tumor | 6 | Low | 1 | 73 | T3 | N0 | M0 | II |
| F09 | Tumor | 12 | High | 1 | 73 | T3 | N0 | M0 | II |
| F11 | Tumor | 8 | High | 1 | 73 | T3 | N0 | M0 | II |
| F13 | Tumor | 8 | High | 0 | 7 | T3 | N1a | M0 | III |
| F15 | Tumor | 7.5 | High | 1 | 73 | T3 | N1a | M0 | III |
| F17 | Tumor | 6 | Low | 1 | 73 | T2 | N0 | M0 | I |
| G01 | Tumor | 8 | Low | 0 | 16 | T4b | N1b | M0 | III |
| G03 | Tumor | 12 | High | 0 | 16 | T4a | N2a | M1a | IV |
| G05 | Tumor | 9 | High | 1 | 72 | T3 | N0 | M0 | II |
| G07 | Tumor | 12 | High | 0 | 21 | T4a | N0 | M0 | II |
| G09 | Tumor | 9 | High | 1 | 72 | T3 | N0 | M0 | II |
| G11 | Tumor | 6 | Low | 1 | 72 | T4a | N0 | M0 | II |
| G13 | Tumor | 8 | High | 1 | 72 | T4a | N1a | M0 | III |
| G15 | Tumor | 7 | Low | 1 | 71 | T3 | N0 | M0 | II |
| G17 | Tumor | 6 | Low | 1 | 71 | T3 | N0 | M0 | II |
| H01 | Tumor | 12 | High | 1 | 71 | T4a | N0 | M0 | II |
| H03 | Tumor | 9 | High | 1 | 71 | T3 | N1a | M0 | III |
| H05 | Tumor | 4 | Low | 1 | 71 | T2 | N0 | M0 | I |
| H07 | Tumor | 9 | High | 1 | 71 | T4a | N0 | M0 | II |
| H09 | Tumor | 9 | High | 1 | 71 | T3 | N0 | M0 | II |
| H11 | Tumor | 7 | Low | 0 | 0.4 | T3 | N2b | M0 | III |
| H13 | Tumor | 6 | Low | 0 | 67 | T3 | N0 | M0 | II |
| H15 | Tumor | 6 | Low | 1 | 70 | T4a | N0 | M0 | II |
| H17 | Tumor | 12 | High | 0 | 58 | T3 | N0 | M0 | II |
| I01 | Tumor | 6 | Low | 1 | 70 | T3 | N0 | M0 | II |
| I03 | Tumor | 5 | Low | 1 | 70 | T3 | N0 | M0 | II |
| I05 | Tumor | 6 | Low | 1 | 70 | T4a | N0 | M0 | II |
| I07 | Tumor | 12 | High | 1 | 70 | T3 | N0 | M0 | II |
| I09 | Tumor | 5 | Low | 1 | 70 | T4b | N0 | M0 | II |
| I11 | Tumor | 1.5 | Low | 0 | 19 | T4a | N2a | M0 | III |
| I13 | Tumor | 8 | High | 0 | 35 | T3 | N0 | M0 | II |
| I15 | Tumor | 8 | High | 1 | 69 | T4a | N1a | M0 | III |
| I17 | Tumor | 2 | Low | 1 | 69 | T4a | N1b | M0 | III |
| J01 | Tumor | 7.5 | High | 1 | 69 | T2 | N0 | M0 | I |
| J03 | Tumor | 8.5 | High | 0 | 42 | T3 | N2b | M0 | III |
| J05 | Tumor | 12 | High | 0 | 19 | T3 | N2a | M0 | III |
| J07 | Tumor | 9 | High | 1 | 69 | T3 | N1b | M0 | III |
| J09 | Tumor | 12 | High | 0 | 15 | T3 | N1b | M0 | III |
| J11 | Tumor | 8.5 | High | 0 | 67 | T4a | N1b | M0 | III |
| J13 | Tumor | 6 | Low | 1 | 69 | T2 | N0 | M0 | I |
| J15 | Tumor | 12 | High | 0 | 23 | T4a | N1b | M0 | III |
| J17 | Tumor | 6 | Low | 0 | 19 | T3 | N1a | M0 | III |

# Table S7 colorectal cancer tissue microarray information.

| Sit location | Tissue Types | Score |
|---|---|---|
| A16 | Normal | null |
| B08 | Normal | null |
| D08 | Normal | null |
| D10 | Normal | null |
| E04 | Normal | null |
| E10 | Normal | null |
| F12 | Normal | null |
| I18 | Normal | null |
| J18 | Normal | null |
| A02 | Normal | 3 |
| A04 | Normal | 1.5 |
| A06 | Normal | 2 |
| A08 | Normal | 1 |
| A10 | Normal | 1 |
| A12 | Normal | 2 |
| A14 | Normal | 1 |
| A18 | Normal | 1.5 |
| B02 | Normal | 2 |
| B04 | Normal | 2 |
| B06 | Normal | 3 |
| B10 | Normal | 8 |
| B12 | Normal | 3 |
| B14 | Normal | 2 |
| B16 | Normal | 1 |
| B18 | Normal | 2 |
| C02 | Normal | 1.5 |
| C04 | Normal | 1.5 |
| C06 | Normal | 3.5 |
| C08 | Normal | 3 |
| C10 | Normal | 4 |
| C12 | Normal | 1 |
| C14 | Normal | 4 |
| C16 | Normal | 3 |
| C18 | Normal | 1.5 |
| D02 | Normal | 2 |
| D04 | Normal | 1 |
| D06 | Normal | 2 |
| D12 | Normal | 1 |
| D14 | Normal | 6 |
| D16 | Normal | 4 |
| D18 | Normal | 2 |
| E02 | Normal | 2 |
| E06 | Normal | 1.5 |
| E08 | Normal | 2 |
| E12 | Normal | 1.5 |
| E14 | Normal | 1 |
| E16 | Normal | 2 |
| E18 | Normal | 1 |
| F02 | Normal | 2 |
| F04 | Normal | 6 |
| F06 | Normal | 2 |
| F08 | Normal | 2 |
| F10 | Normal | 6 |
| F14 | Normal | 1.5 |
| F16 | Normal | 2 |
| F18 | Normal | 2 |
| G02 | Normal | 4 |
| G04 | Normal | 8 |
| G06 | Normal | 4 |
| G08 | Normal | 1 |
| G10 | Normal | 4 |
| G12 | Normal | 2 |
| G14 | Normal | 2 |
| G16 | Normal | 1.5 |
| G18 | Normal | 1.5 |
| H02 | Normal | 1.5 |
| H04 | Normal | 4 |
| H06 | Normal | 8 |
| H08 | Normal | 6 |
| H10 | Normal | 6 |
| H12 | Normal | 2 |
| H14 | Normal | 2 |
| H16 | Normal | 3 |
| H18 | Normal | 6 |
| I02 | Normal | 6 |
| I04 | Normal | 2 |
| I06 | Normal | 3 |
| I08 | Normal | 1.5 |
| I10 | Normal | 1.5 |
| I12 | Normal | 1.5 |
| I14 | Normal | 1.5 |
| I16 | Normal | 2 |
| J02 | Normal | 1.5 |
| J04 | Normal | 3 |
| J06 | Normal | 2.5 |
| J08 | Normal | 1.5 |
| J10 | Normal | 3 |
| J12 | Normal | 2 |
| J14 | Normal | 1.5 |
| J16 | Normal | 2 |

# Table S8 lactylation-related genes

| genes | | | | |
|---|---|---|---|---|
| ACTN3 | ARTN | EXT2 | MDH1 | SAP30 |
| GATD1 | AURKA | FAM162A | MDH2 | SDC1 |
| HAGH | B3GALT6 | FBP2 | ME1 | SDC2 |
| HIF1A | B3GAT1 | FKBP4 | ME2 | SDC3 |
| LDHA | B3GAT3 | FUT8 | MED24 | SDHC |
| LDHAL6A | B3GNT3 | G6PD | MERTK | SLC25A10 |
| LDHAL6B | B4GALT1 | GAL3ST1 | MET | SLC25A13 |
| LDHB | B4GALT2 | GALE | MIF | SLC35A3 |
| LDHC | B4GALT4 | GALK1 | MIOX | SLC37A4 |
| LDHD | B4GALT7 | GALK2 | MPI | SOD1 |
| MIR210 | BIK | GAPDHS | MXI1 | SOX9 |
| MRS2 | BPNT1 | GCLC | NANP | SPAG4 |
| PARK7 | CACNA1H | GFPT1 | NASP | SRD5A3 |
| PER2 | CAPN5 | GFUS | NDST3 | STC1 |
| PFKFB2 | CASP6 | GLCE | NDUFV3 | STC2 |
| PNKD | CD44 | GLRX | NOL3 | STMN1 |
| TIGAR | CDK1 | GMPPA | NSDHL | TALDO1 |
| TP53 | CENPA | GMPPB | NT5E | TFF3 |
| ACACB | CHPF | GNE | P4HA1 | TGFA |
| EMB | CHPF2 | GNPDA1 | P4HA2 | TGFBI |
| SLC16A1 | CHST1 | GOT1 | PAM | TKTL1 |
| SLC16A3 | CHST12 | GOT2 | PAXIP1 | TPBG |
| SLC16A7 | CHST2 | GPC1 | PC | TPI1 |
| SLC16A8 | CHST4 | GPC3 | PDK3 | TPST1 |
| SLC5A12 | CHST6 | GPC4 | PFKFB1 | TXN |
| SLC5A8 | CITED2 | GPR87 | PFKP | UGP2 |
| EP300 | CLDN3 | GUSB | PGAM1 | VCAN |
| CREBBP | CLDN9 | GYS1 | PGAM2 | VEGFA |
| KAT5 | CLN6 | GYS2 | PGK1 | VLDLR |
| AARS2 | COG2 | HAX1 | PGLS | XYLT2 |
| AARS1 | COL5A1 | HDLBP | PGM2 | ZNF292 |
| KAT8 | COPB2 | HK2 | PHKA2 | |
| KAT7 | CTH | HMMR | PKM | |
| ATAT1 | CXCR4 | HOMER1 | PKP2 | |
| ABCB6 | CYB5A | HS2ST1 | PLOD1 | |
| ADORA2B | DCN | HS6ST2 | PLOD2 | |
| AGL | DDIT4 | HSPA5 | PMM2 | |
| AGRN | DEPDC1 | IDH1 | POLR3K | |
| AK3 | DLD | IDUA | PPFIA4 | |
| AK4 | DPYSL4 | IER3 | PPIA | |
| AKR1A1 | DSC2 | IGFBP3 | PPP2CB | |
| ALDH7A1 | ECD | IL13RA1 | PRPS1 | |
| ALDH9A1 | EFNA3 | IRS2 | PSMC4 | |
| ALDOA | EGFR | ISG20 | PYGB | |
| ALDOB | EGLN3 | KDELR3 | PYGL | |
| ALG1 | ELF3 | KIF20A | QSOX1 | |
| ANG | ENO1 | KIF2A | RARS1 | |
| ANGPTL4 | ENO2 | LCT | RBCK1 | |
| ANKZF1 | ERO1A | LHPP | RPE | |
| ARPP19 | EXT1 | LHX9 | RRAGD | |
