# Supplementary material for: Histone lactylation enhances GCLC expression and thus promotes chemoresistance of colorectal cancer stem cells through inhibiting ferroptosis
Source: Cell Death Dis. 2025 Mar 20;16(1):193. doi: 10.1038/s41419-025-07498-z (PMC11926133; doi:10.1038/s41419-025-07498-z)

Fig.1

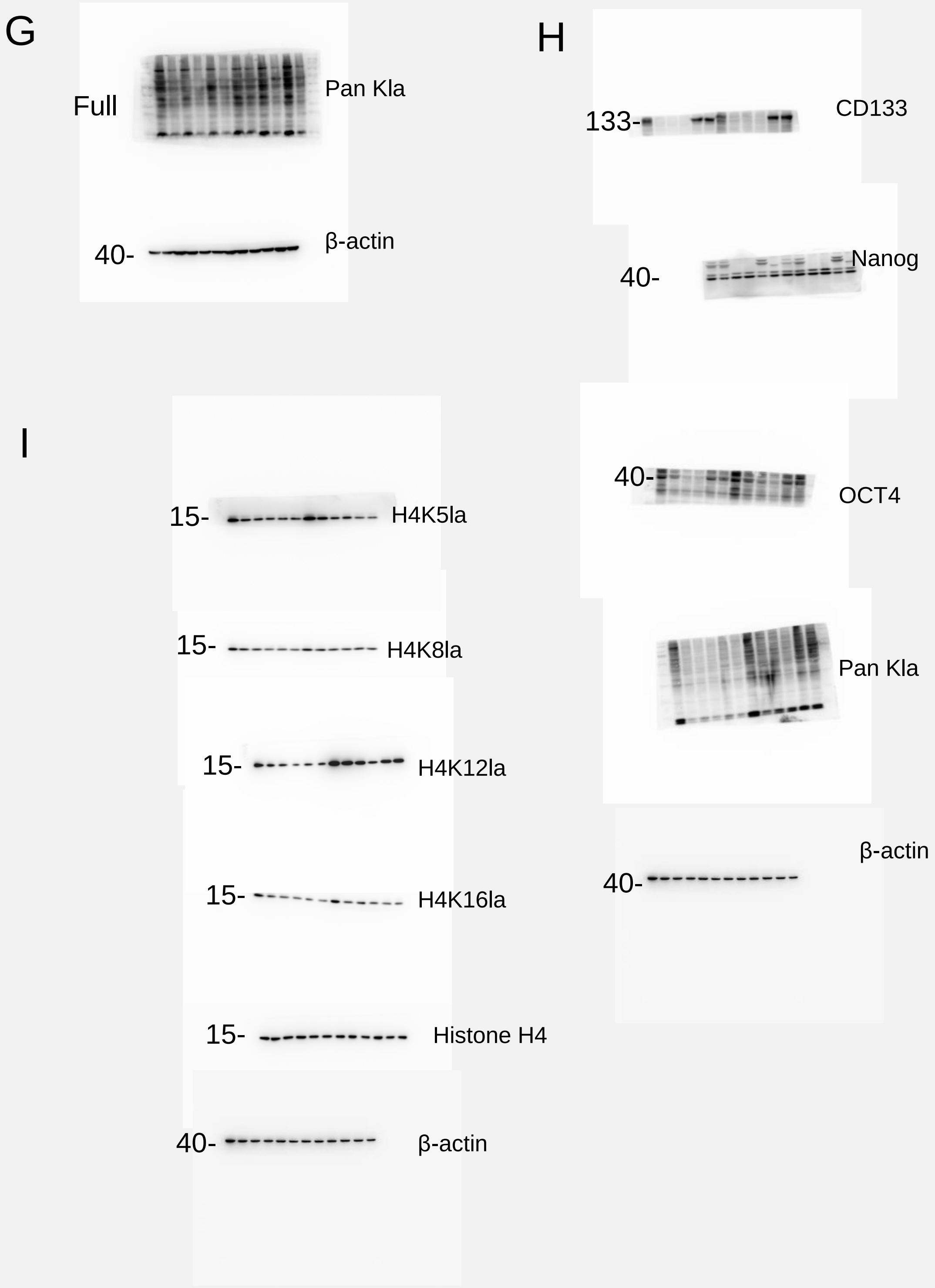
G
Full
Pan Kla
40-
β-actin
H
133-
CD133
40-
Nanog
40-
OCT4
Pan Kla
40-
β-actin
I
15-
H4K5la
15-
H4K8la
15-
H4K12la
15-
H4K16la
15-
Histone H4
40-
β-actin

Fig.2

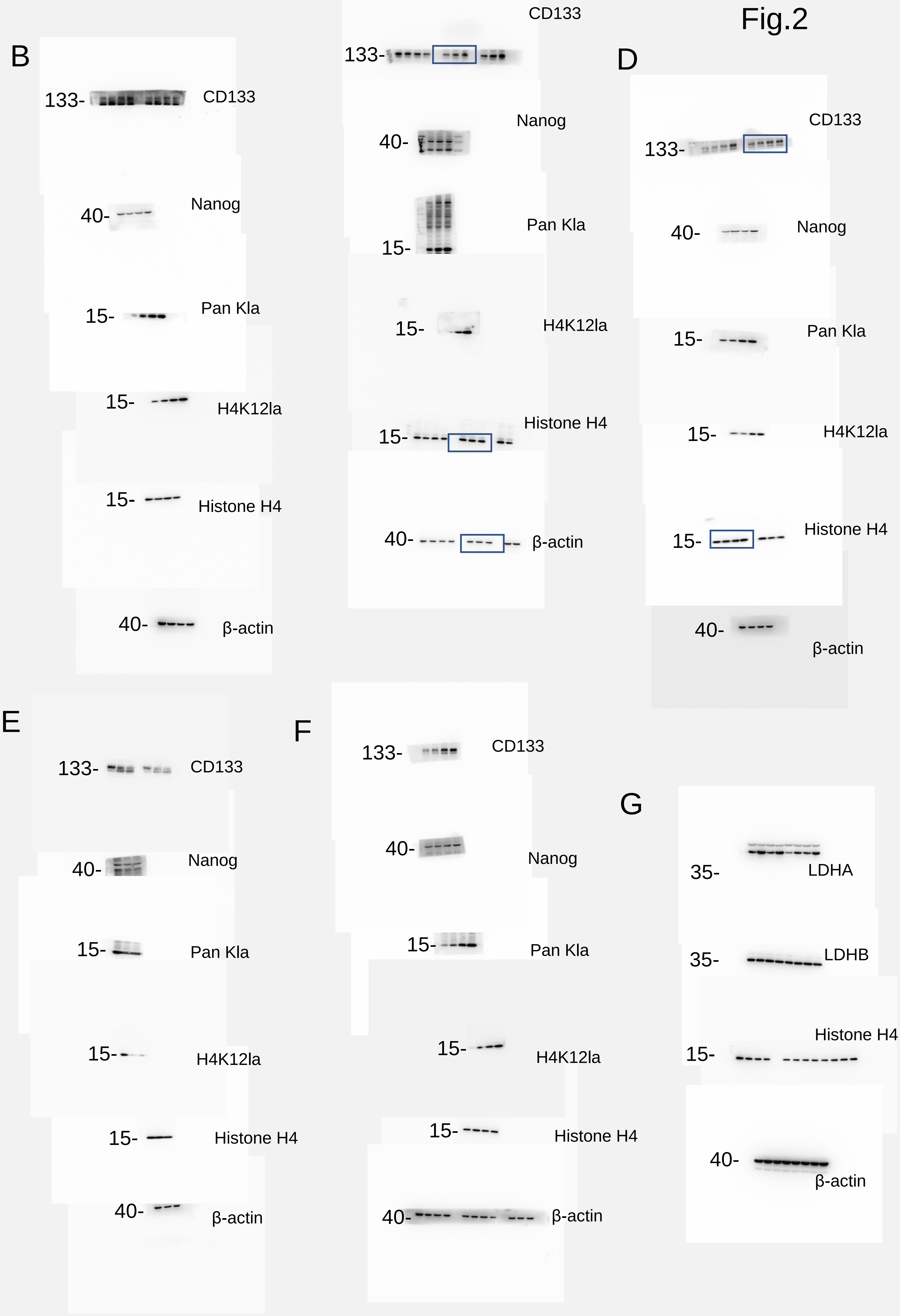

B
133-
CD133
40-
Nanog
15-
Pan Kla
15-
H4K12la
15-
Histone H4
40-
β-actin
CD133
133-
Nanog
40-
Pan Kla
15-
15-
H4K12la
Histone H4
15-
40-
β-actin
D
CD133
133-
40-
Nanog
15-
Pan Kla
15-
H4K12la
Histone H4
15-
40-
β-actin
E
133-
CD133
40-
Nanog
15-
Pan Kla
15-
H4K12la
15-
Histone H4
40-
β-actin
F
133-
CD133
40-
Nanog
15-
Pan Kla
15-
H4K12la
15-
Histone H4
40-
β-actin
G
35-
LDHA
35-
LDHB
Histone H4
15-
40-
β-actin

Fig.2

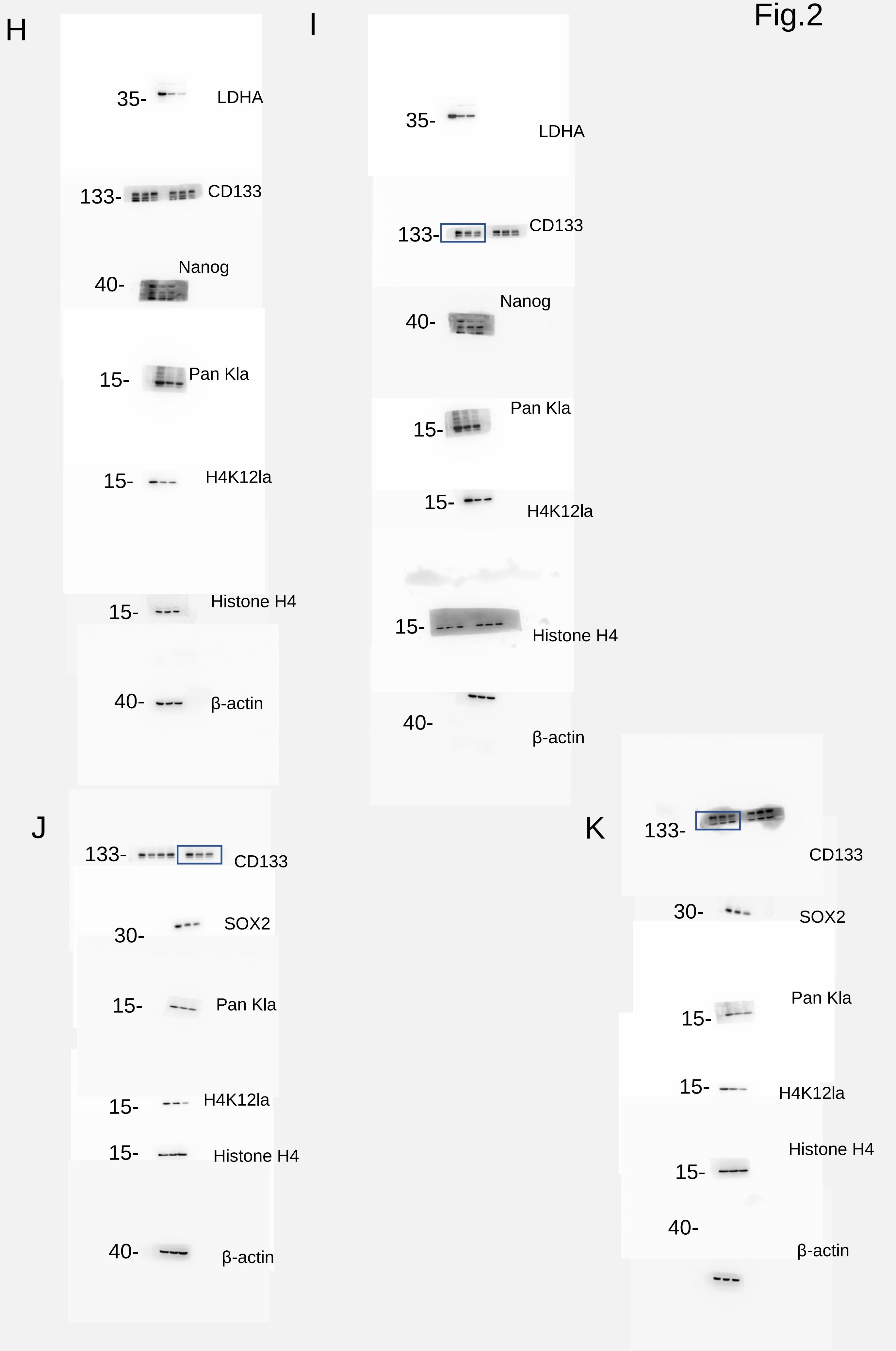
H
35-
LDHA
133-
CD133
Nanog
40-
15-
Pan Kla
15-
H4K12la
15-
Histone H4
40-
β-actin
I
35-
LDHA
133-
CD133
Nanog
40-
Pan Kla
15-
15-
H4K12la
15-
Histone H4
40-
β-actin
J
133-
CD133
30-
SOX2
15-
Pan Kla
15-
H4K12la
15-
Histone H4
40-
β-actin
K
133-
CD133
30-
SOX2
Pan Kla
15-
15-
H4K12la
Histone H4
15-
40-
β-actin

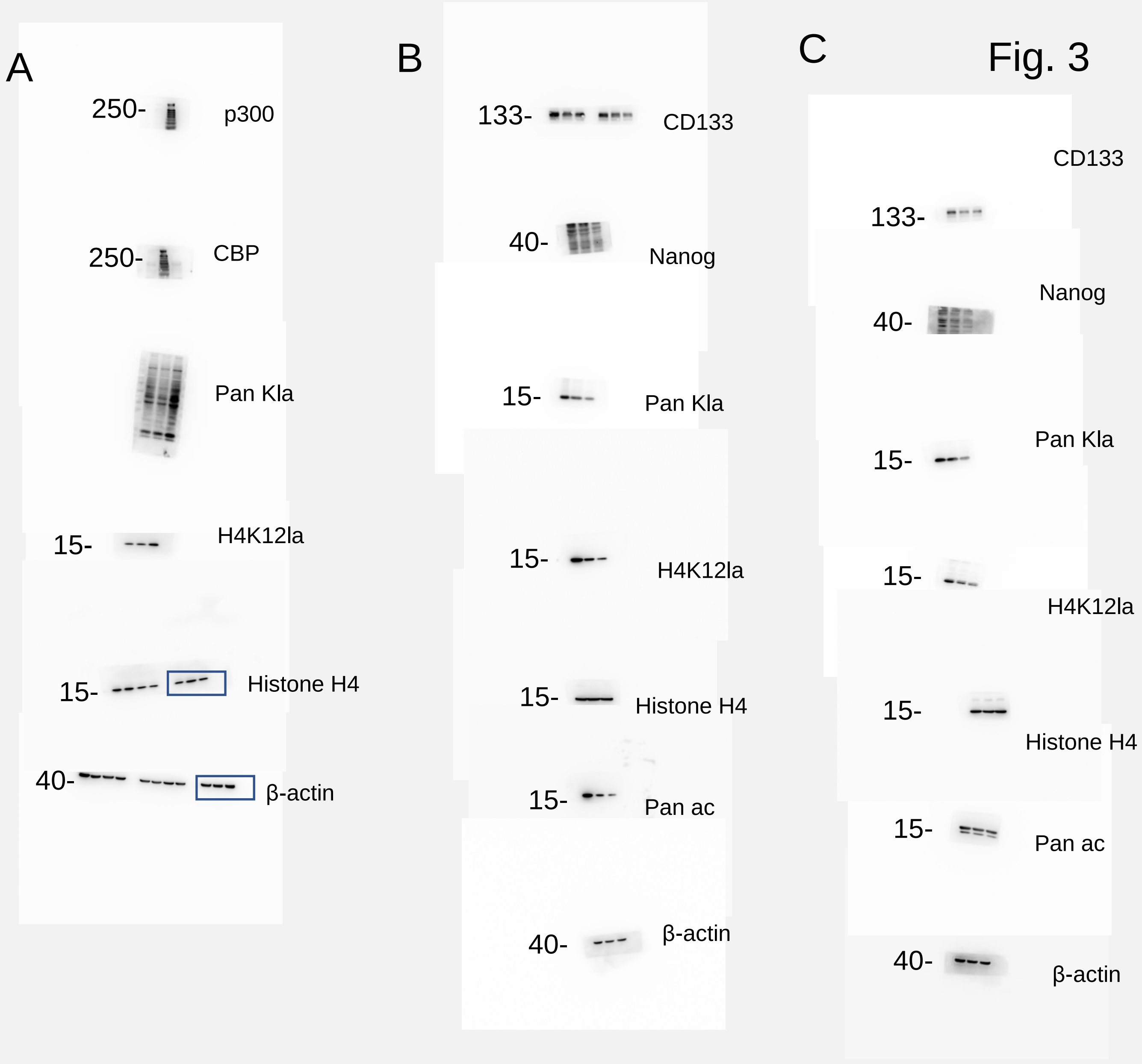
A
250-
p300
250-
CBP
Pan Kla
15-
H4K12la
15-
Histone H4
40-
β-actin
B
133-
CD133
40-
Nanog
15-
Pan Kla
15-
H4K12la
15-
Histone H4
15-
Pan ac
40-
β-actin
C
CD133
133-
Nanog
40-
15-
Pan Kla
15-
H4K12la
15-
Histone H4
15-
Pan ac
40-
β-actin
Fig. 3

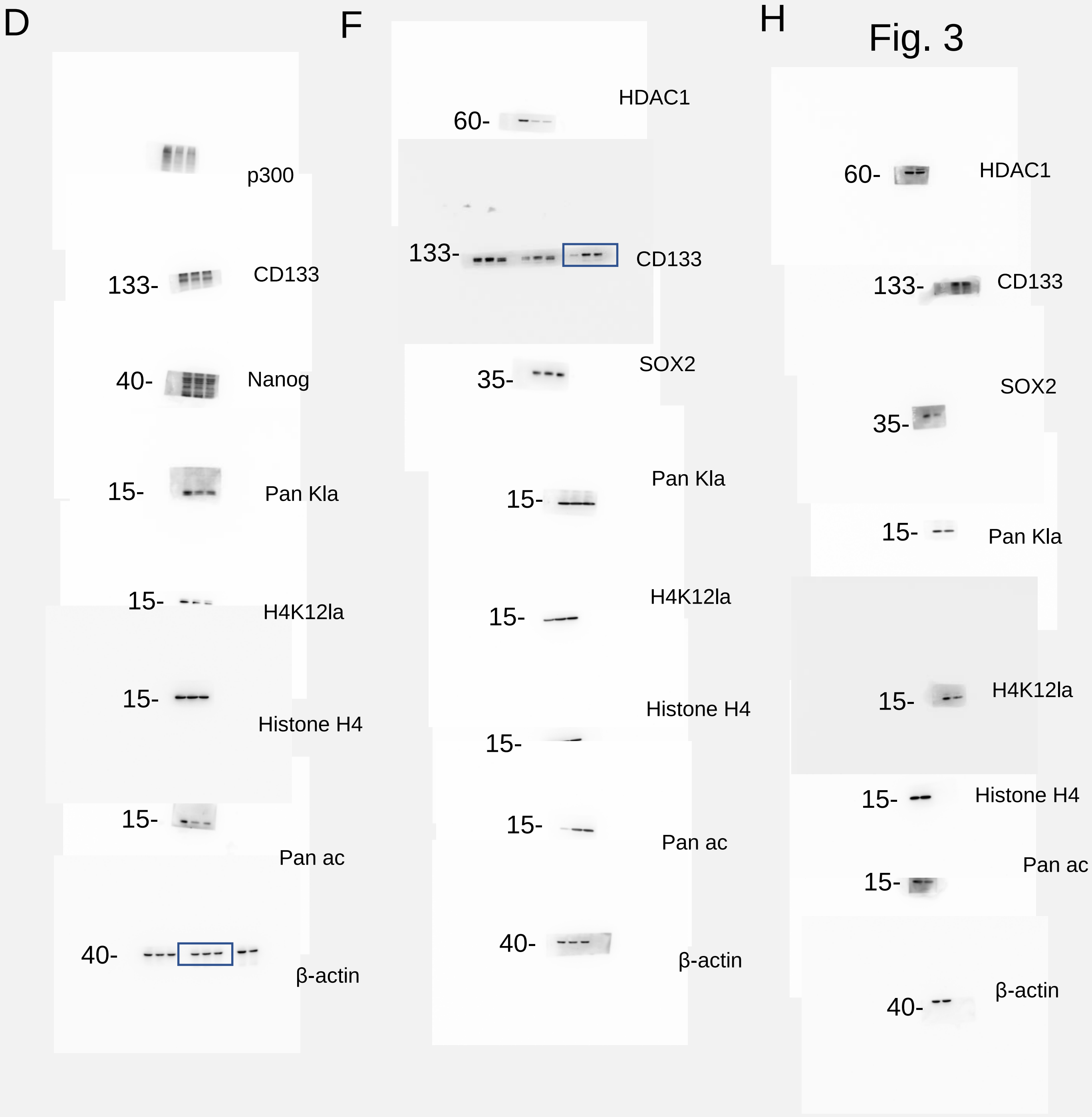
D
F
H
Fig. 3
p300
133-
CD133
40-
Nanog
15-
Pan Kla
15-
H4K12la
15-
Histone H4
15-
Pan ac
40-
β-actin
60-
HDAC1
133-
CD133
35-
SOX2
15-
Pan Kla
15-
H4K12la
Histone H4
15-
15-
Pan ac
40-
β-actin
60-
HDAC1
133-
CD133
SOX2
35-
15-
Pan Kla
15-
H4K12la
15-
Histone H4
15-
Pan ac
40-
β-actin

Fig.6

F

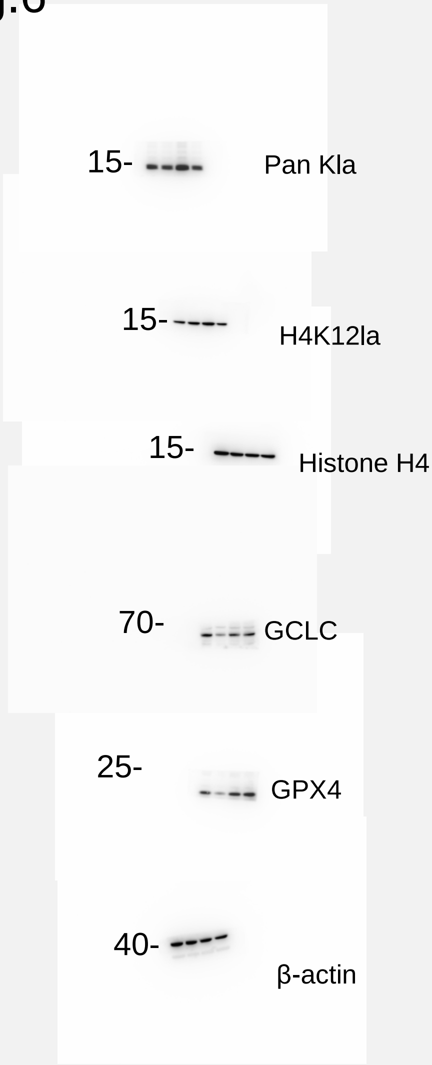
15- Pan Kla
15- H4K12la
15- Histone H4
70- GCLC
25- GPX4
40- β-actin

I

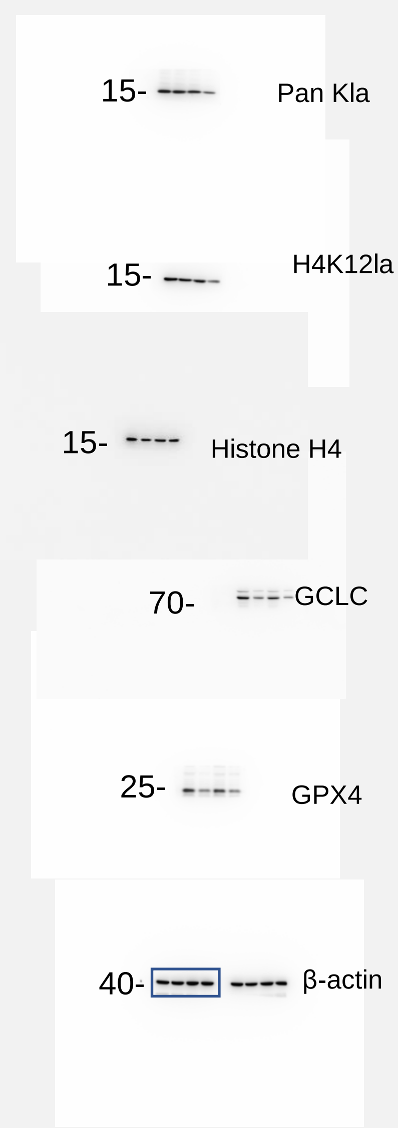
15- Pan Kla
15- H4K12la
15- Histone H4
70- GCLC
25- GPX4
40- β-actin

Fig.7

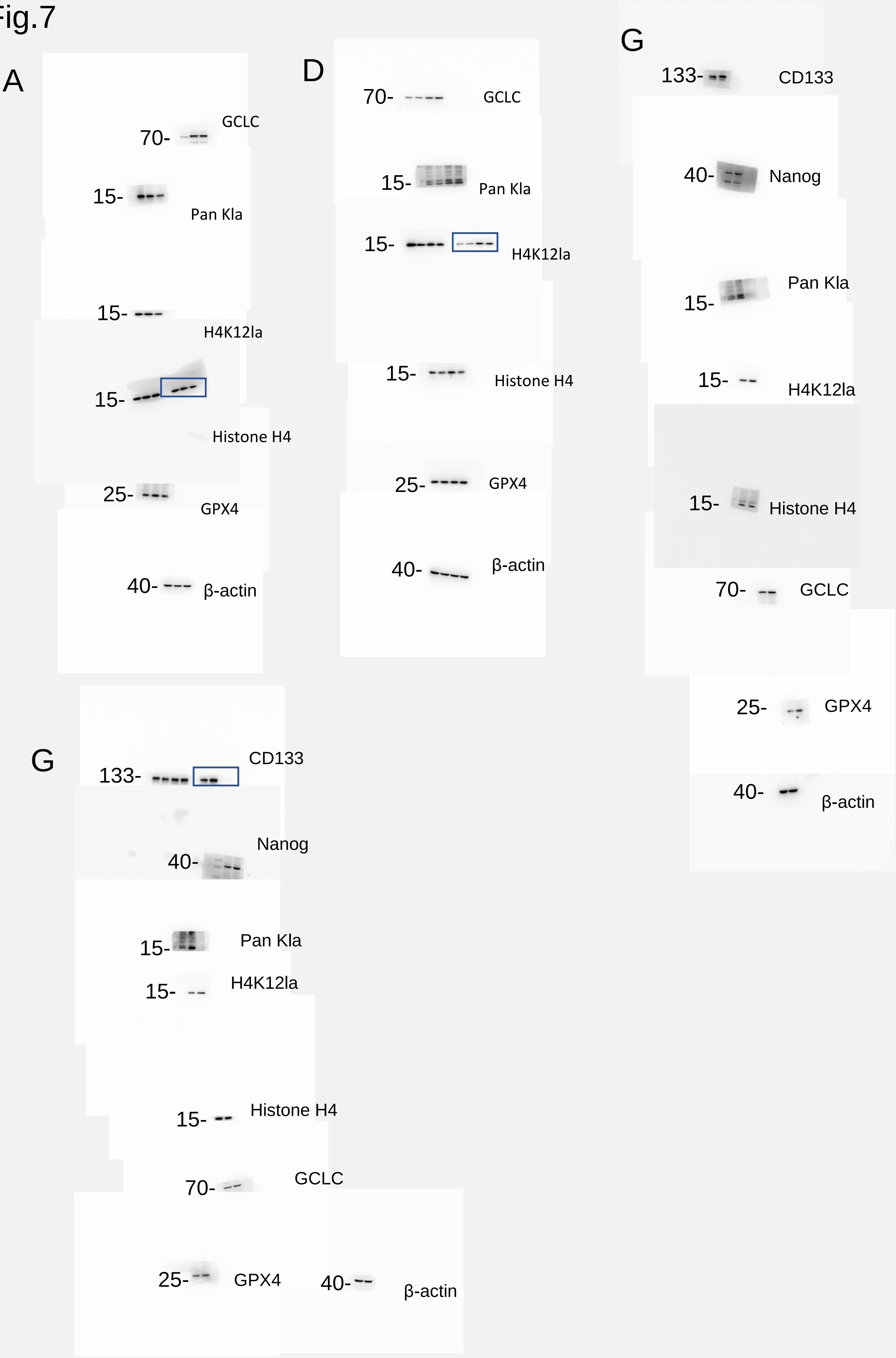
A
70-
GCLC
15-
Pan Kla
15-
H4K12la
15-
Histone H4
25-
GPX4
40-
β-actin
D
70-
GCLC
15-
Pan Kla
15-
H4K12la
15-
Histone H4
25-
GPX4
40-
β-actin
G
133-
CD133
40-
Nanog
15-
Pan Kla
15-
H4K12la
15-
Histone H4
70-
GCLC
25-
GPX4
40-
β-actin
G
133-
CD133
40-
Nanog
15-
Pan Kla
15-
H4K12la
15-
Histone H4
70-
GCLC
25-
GPX4
40-
β-actin

Supplemental Fig.1

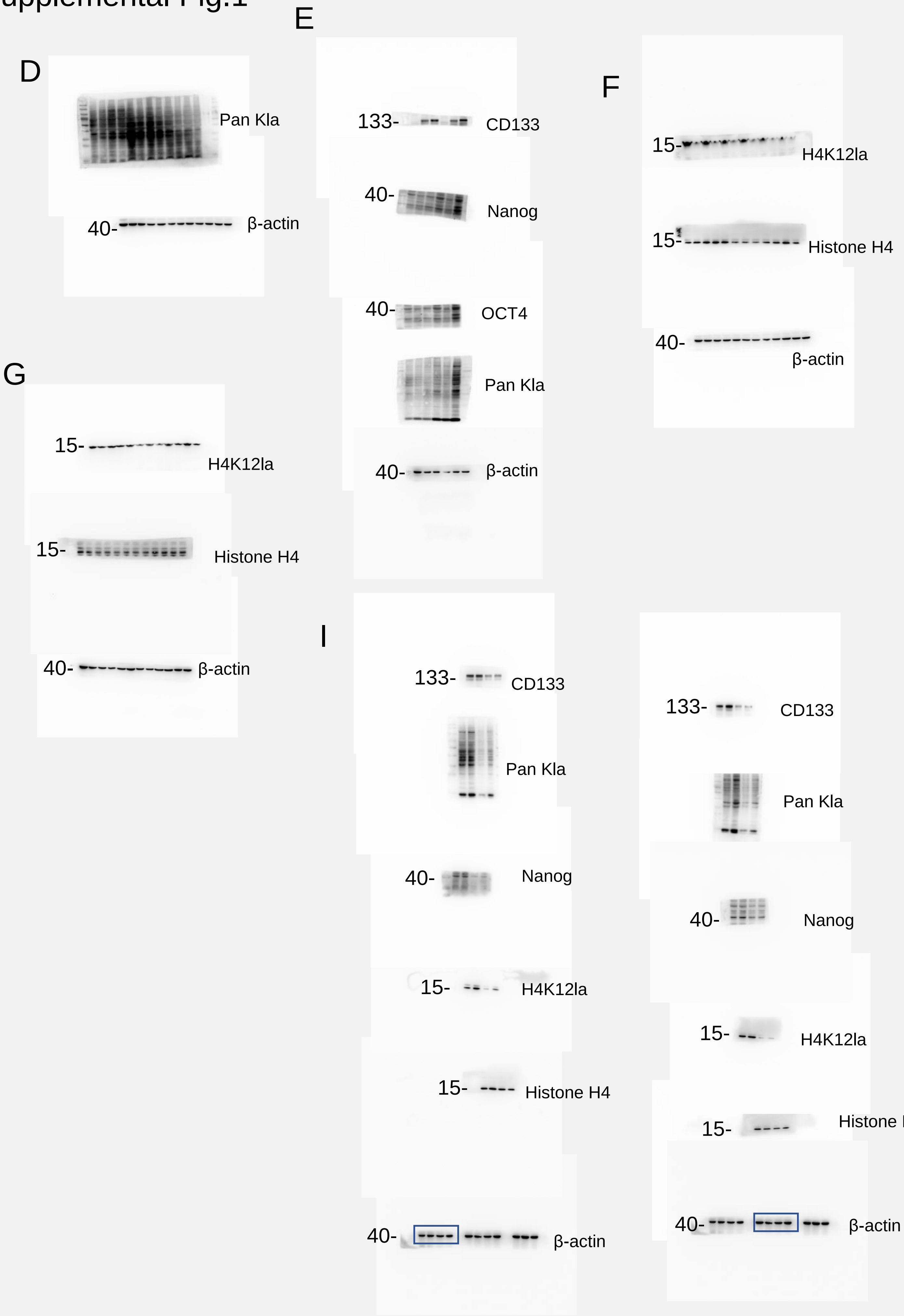

Supplemental Fig.2

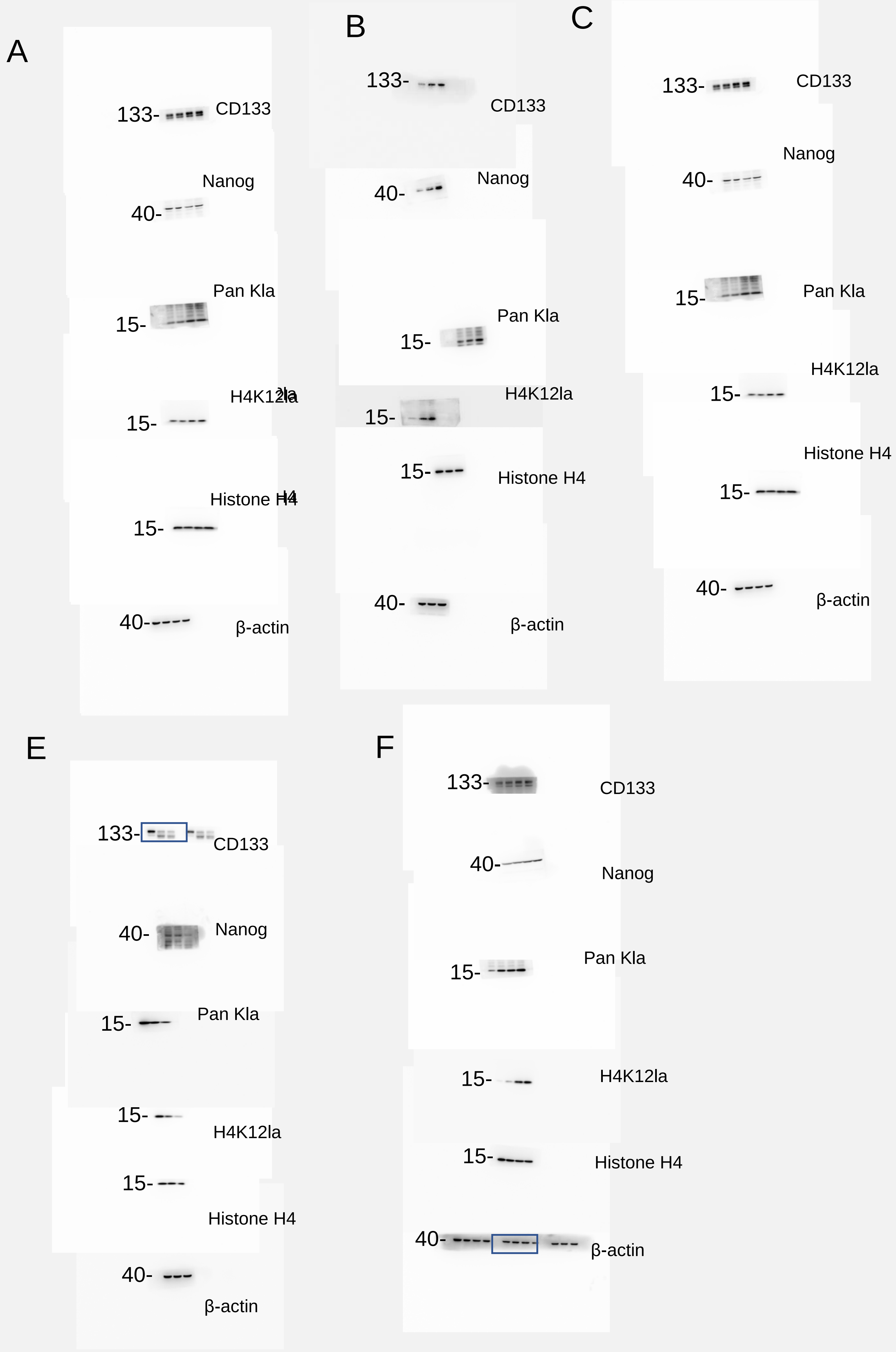

Supplemental Fig.3

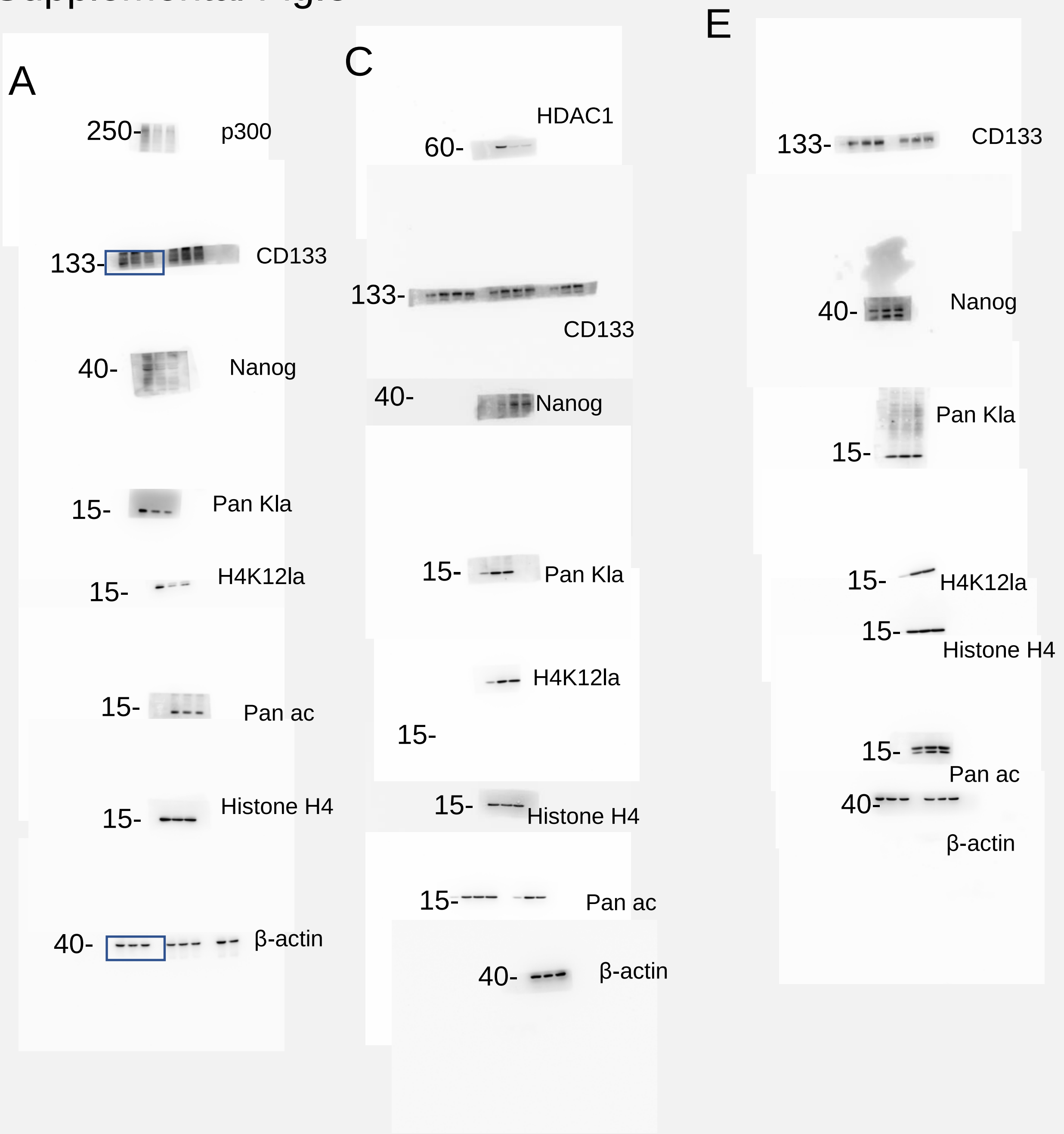

Supplemental Fig.3

E

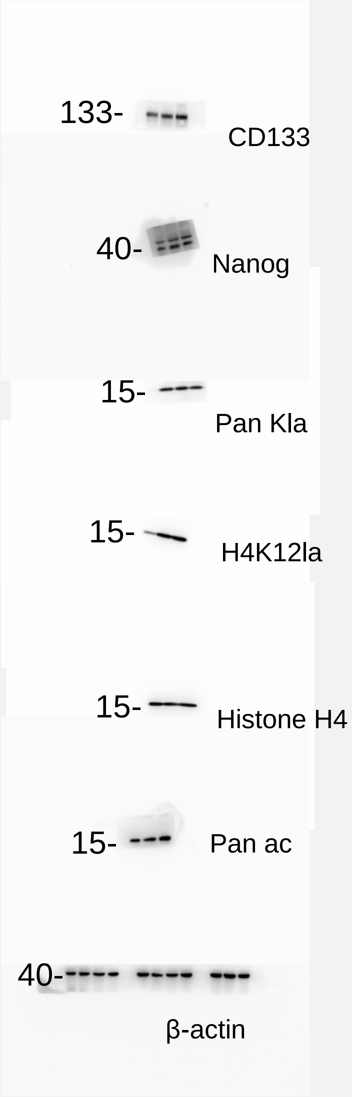

F

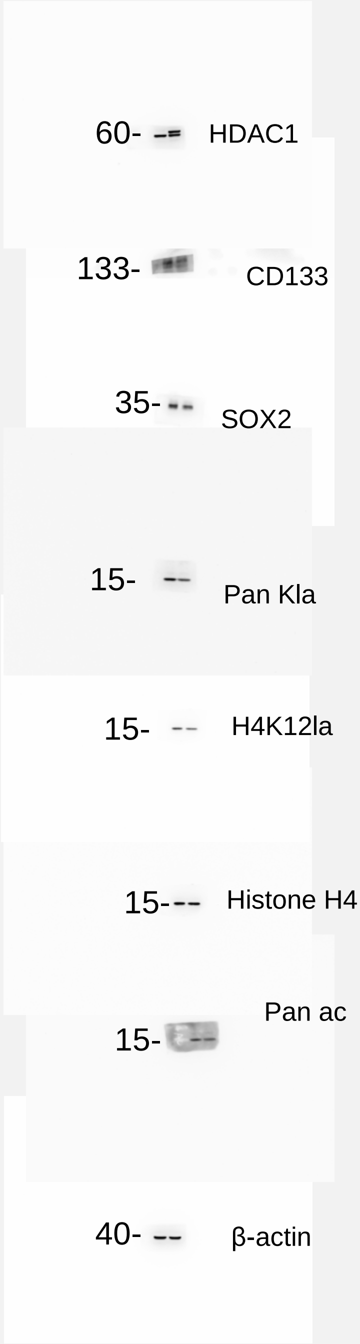

Supplemental Fig.7

F

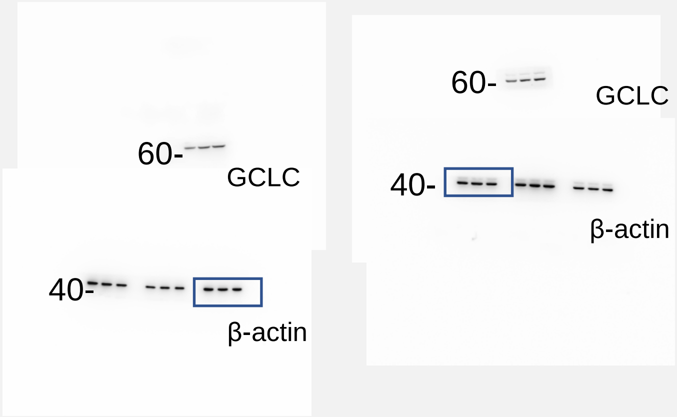

H

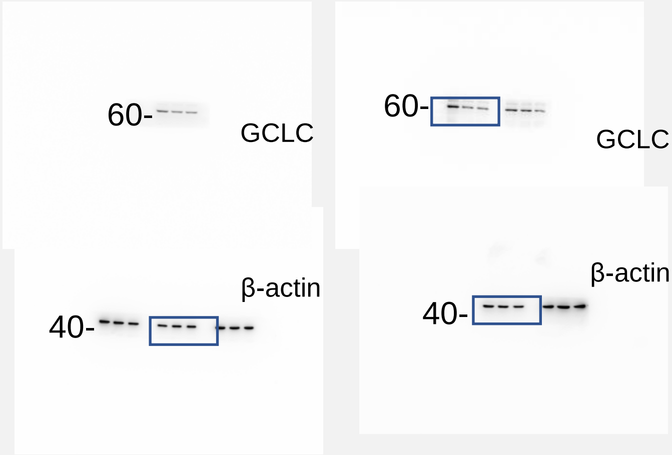

J

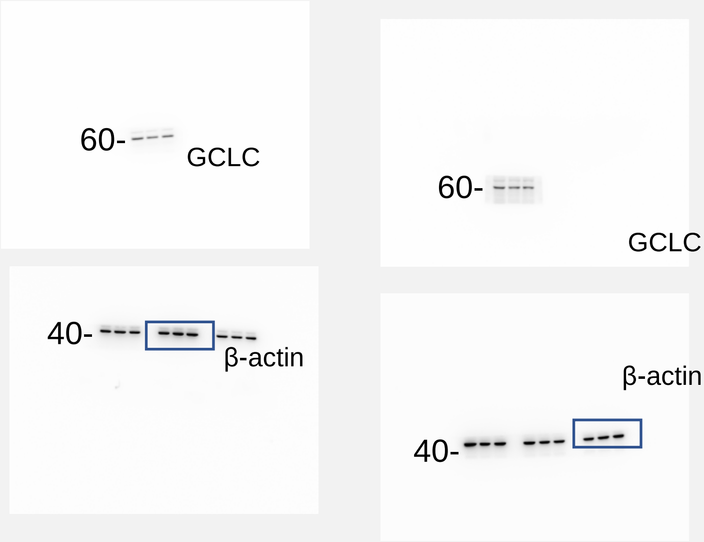

L

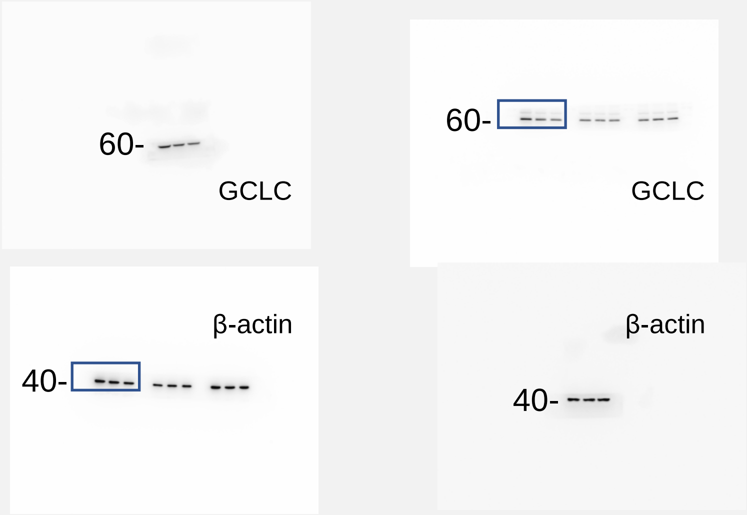

Supplemental Fig.8

A

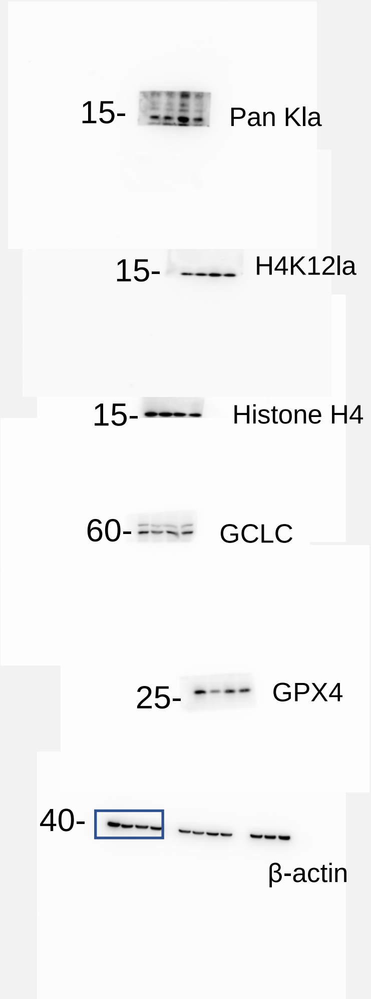

D

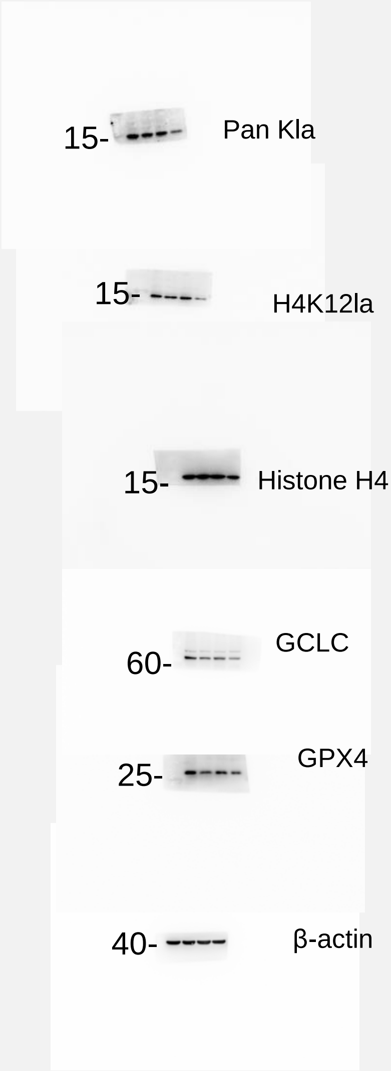

Supplemental Fig.9

A

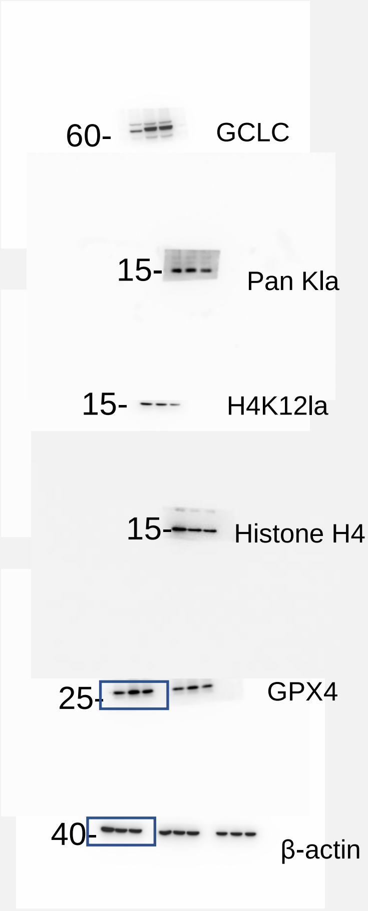

D

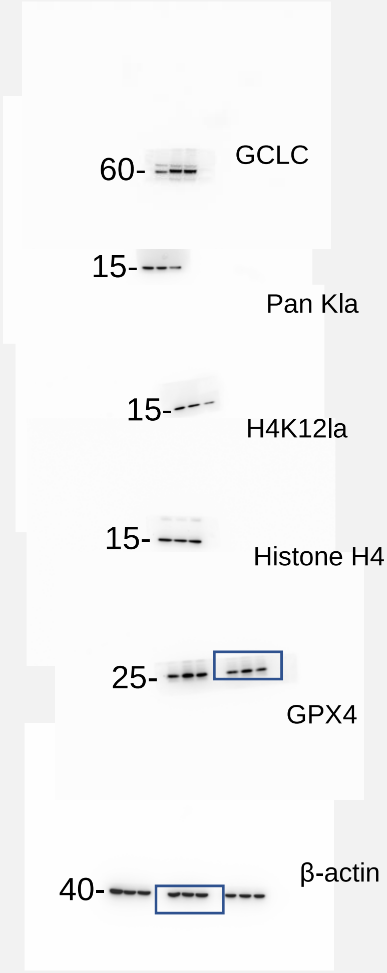

G

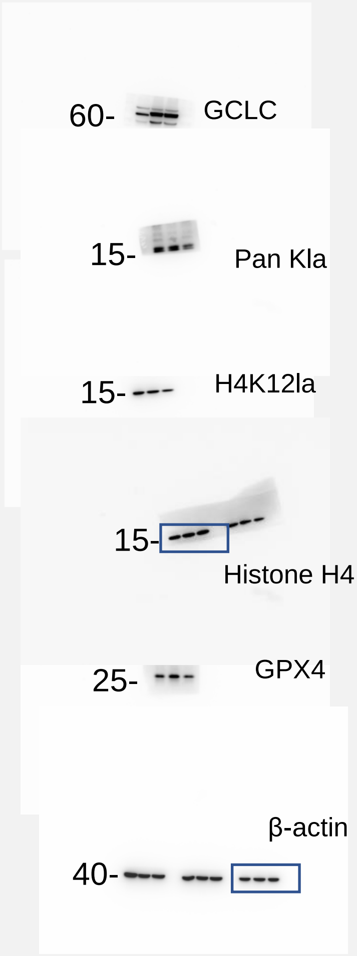

Supplemental Fig. 10

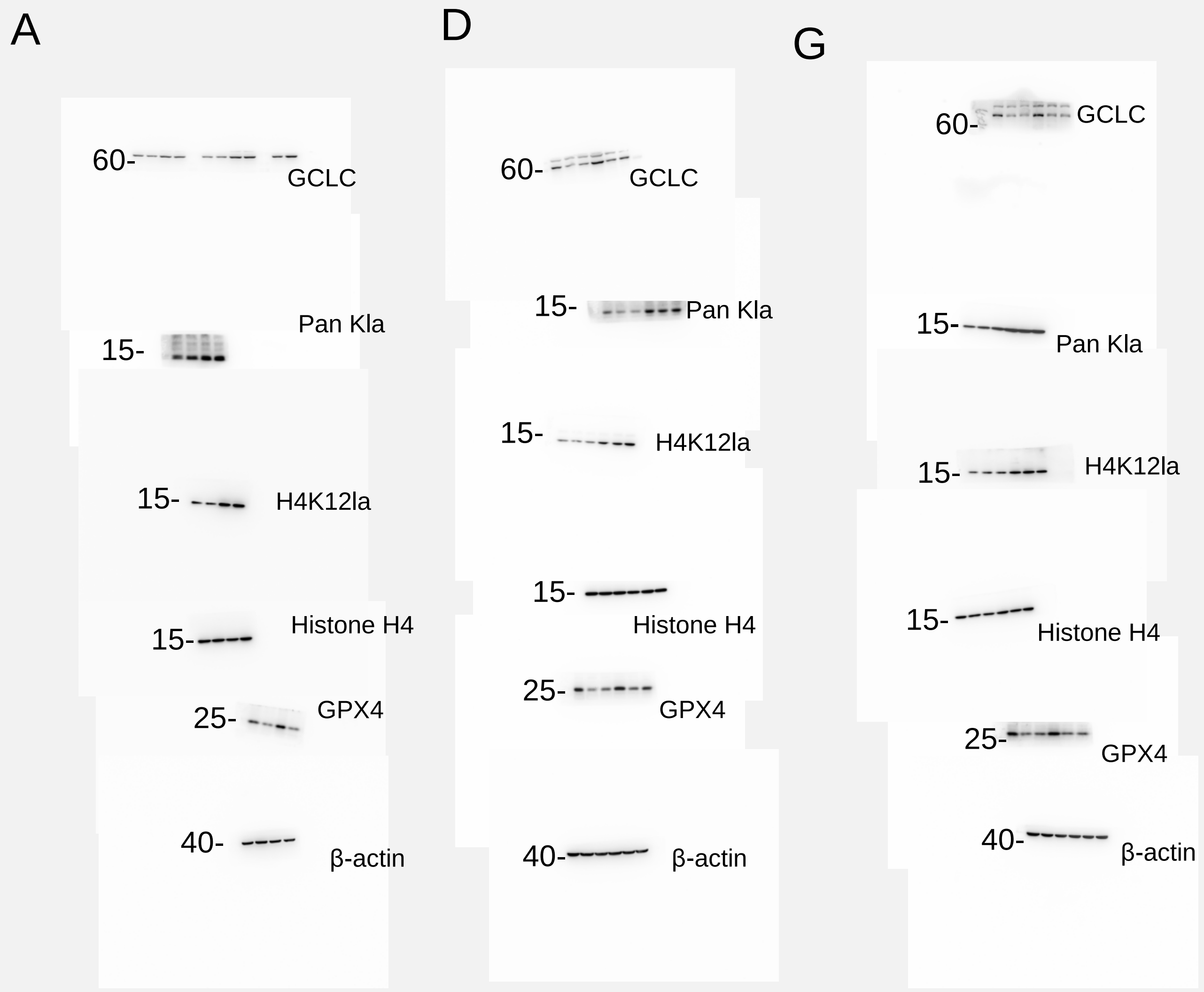

Supplement: Supplementary file 2 — original western blots [file 41419_2025_7498_MOESM2_ESM.pdf]
